# Supplementary material for: Positive Prognostic Overall Survival Impacts of Methylated TGFB2 and MGMT in Adult Glioblastoma Patients
Source: Cancers (Basel). 2025 Mar 27;17(7):1122. doi: 10.3390/cancers17071122 (PMC11987947; doi:10.3390/cancers17071122)
Supplement: Supplementary file 1 [file cancers-17-01122-s001.zip › cancers-3478443-supplementary.pdf]

Supplementary material

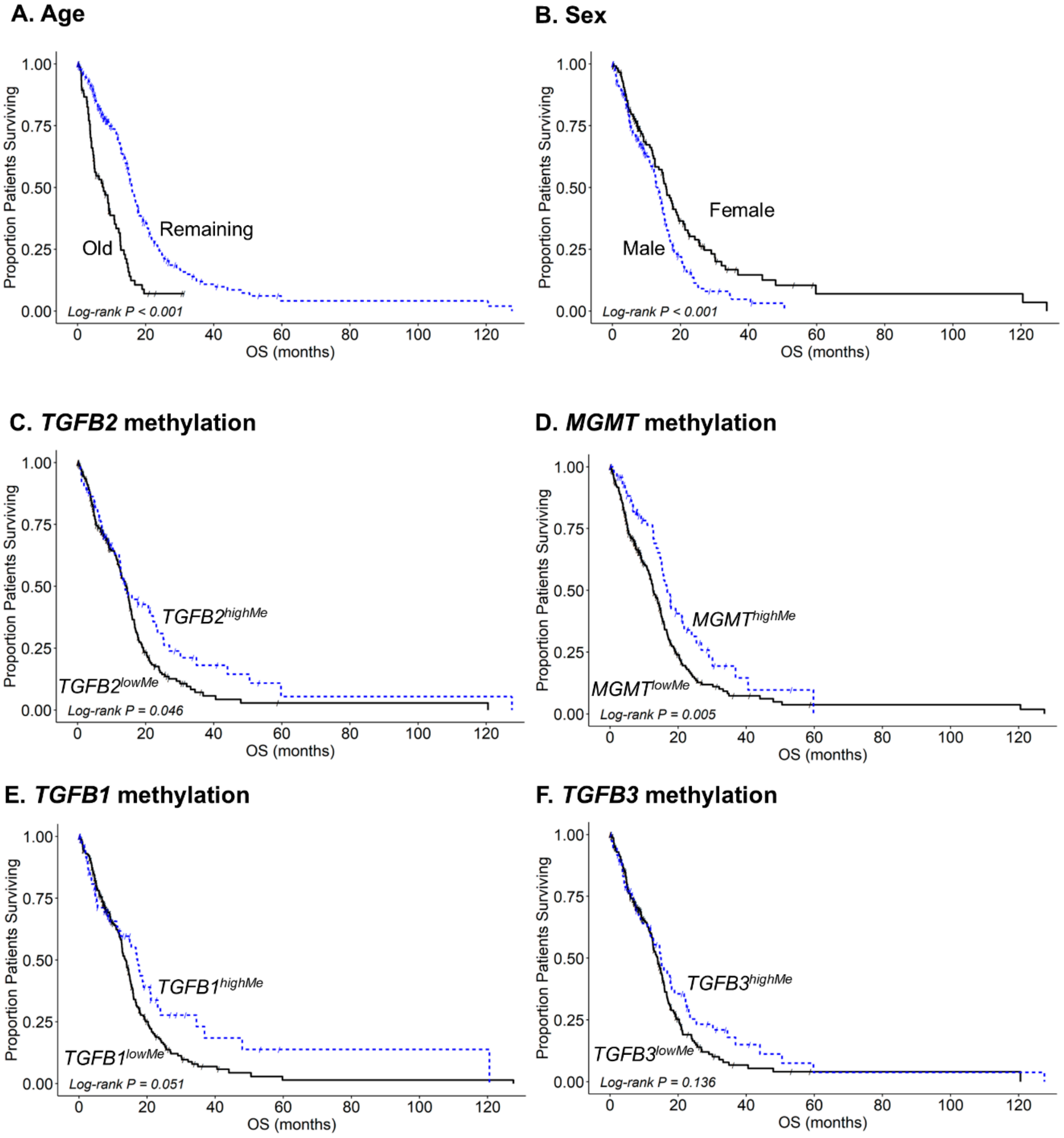

Figure S1. Impact of age and sex on Overall Survival (OS) in GBM Patients.

OS and methylation data from the TCGA resource were available for 292 GBM patients. These patients were stratified into Old and Remaining patients (Cut-off at Upper Quartile of 69 yr) (**A**) and Male versus Female (**B**), and the OS was determined using Kaplan Meier analysis. [**A**] The median OS time for 78 Old patients was 7.63 (95% CI: 4.87 - 10.85, # Death Events = 61) months, which was significantly (Log-rank Chi-Square = 38.64, P-value < 0.001) shorter than the 214 remaining patients (median OS = 15.9 (95% CI: 14.9 - 17.65) months, # Death Events = 146). [**B**] Female patients (N=121, median OS = 15.9 (95% CI: 12.5 - 19.3) months, # Death Events = 82) exhibited a significantly (Log-rank Chi-Square = 38.64, P-value < 0.001) improved survival outcome than Male patients (N=171, median OS = 13 (95% CI: 12.23 - 14.92) months, # Death Events = 127) in this cohort of GBM patients. GBM patients were interrogated using the TCGA dataset and correlated to methylation beta values (25th percentile cut-off for high methylation levels; superscripted "highMe"(N=73) compared to low methylation; "lowMe" (N=219) for each of the genes) to investigate the prognostic impact of **C.** *TGFB2*, **D.** *MGMT*, **E.** *TGFB1*, and **F.** *TGFB3* methylation in these patients. This Kaplan-Meier analysis demonstrated that the survival curves of *TGFB2* methylation significantly diverged after 20 months (P = 0.046) (C). Patients with high levels of *MGMT* methylations exhibited significantly longer median OS time than remaining patients (16.8 compared to 12.7 months, P = 0.005) (D). Patients with high levels of *TGFB1* (E, P = 0.051) and *TGFB3* methylations did not achieve statistical significance for separating the OS curves (F, P = 0.136).

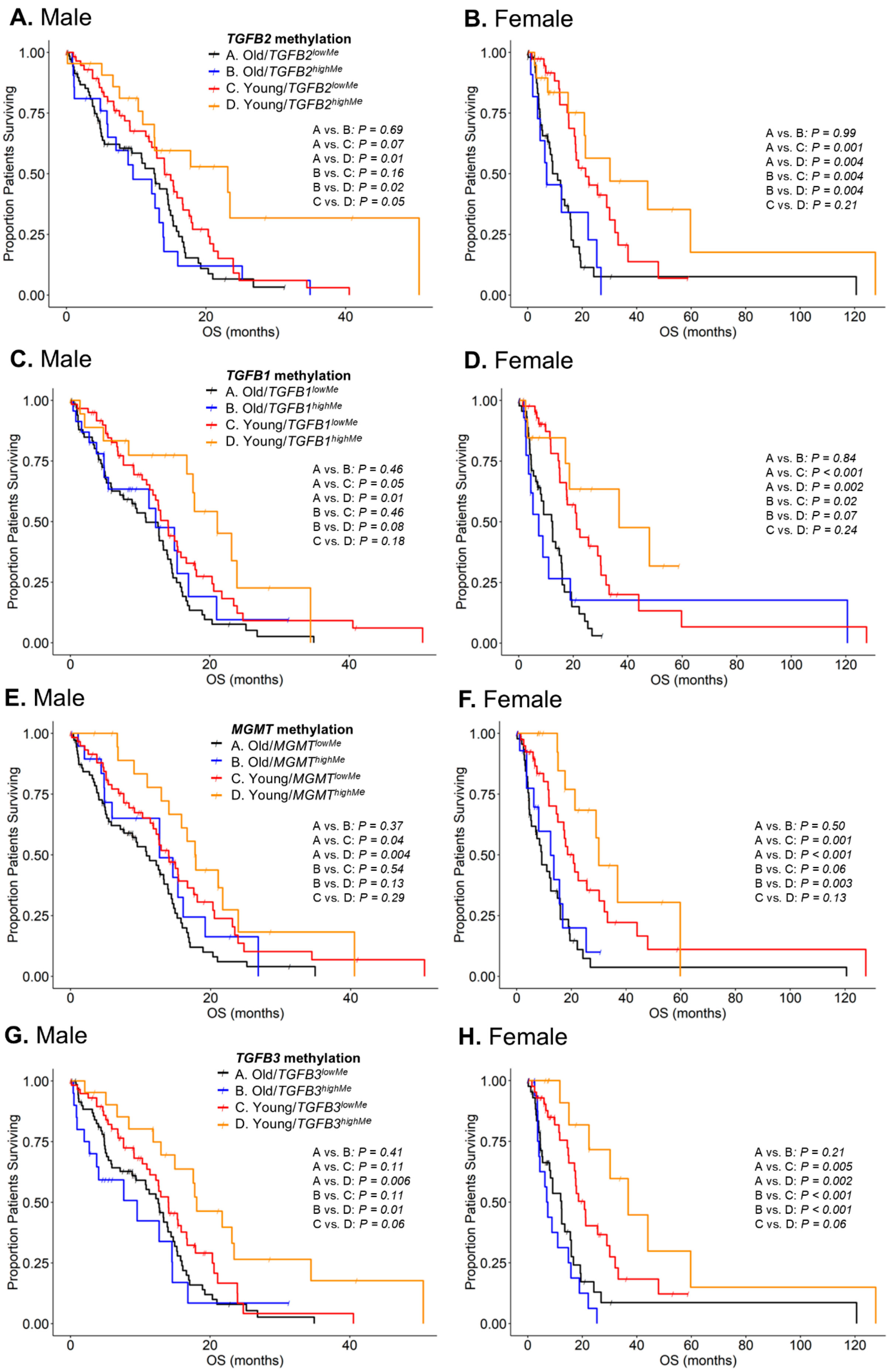

Figure S2. The prognostic OS impact of *TGFB1*/2/3 and *MGMT* gene methylations in adult GBM patients stratified according to age at diagnosis and sex.

GBM patients were interrogated using the TCGA dataset to correlate OS to methylation beta values (25th percentile cut-off for high methylation levels; superscripted "highMe" compared to low methylation; "lowMe" for each of the genes) stratified

according to both sex and age at diagnosis (median cut-off for Old and Young patients = 60 yrs) to investigate the prognostic impact of *TGFB2* (**A, B**) , *TGFB1* (**C, D**) , *MGMT* (**E, F**) , and *TGFB3* (**G, H**) methylation in these patients. The Kaplan Meier plots show curves for Old GBM patients with low methylation levels (black lines), Old patients with high methylation levels (blue lines, Young patients with low methylation levels (red lines), and Young patients with high methylation levels (orange lines). [**A**] The median survival (mOS) time for 70 Old Male patients with low levels of *TGFB2* methylation was 12.7 (95% CI: 7.6 - 14.9, death events = 52) months, which was not statistically different from patients with high levels of *TGFB2* methylation (N=21; mOS = was 9.5 (95% CI: 5.9 - 14, death events = 18; P = 0.69) months. Younger males exhibited improved survival outcomes, whereby 58 Young Male patients with low levels of *TGFB2* methylation experienced mOS of 14.1 (95% CI: 12.2 - 17.6, death events = 43) months, this survival outcome was significantly improved at high levels of *TGFB2* methylation to 23.1 (N = 22; 95% CI: 12.6 - NA, death events = 12) months; P = 0.05). At high levels of *TGFB2* methylation, the survival times for Young and Old males were statistically significant (mOS of 23.1 versus 9.5 months, respectively; P=0.019). [**B**] Old Females exhibited short survival times that were not impacted by *TGFB2* methylation levels (52 *TGFB2*^{lowMe} patients exhibited mOS of 9.2 (95% CI: 8 - 15.6, death events = 38) months, compared to *TGFB*^{highMe} patients for whom mOS was 7 (N = 11; 95% CI: 4.6 - NA, death events = 10) months; P=0.99). Young Female patients experienced pronounced improvements in survival times, but the improvement for *TGFB2*^{highMe} patients did not achieve statistical significance due to low patient numbers post-stratification. 39 Young Female patients with low *TGFB2* methylation experienced mOS of 21.3 (95% CI: 17.5 - 33, death events = 24) months compared to 19 patients experiencing mOS of 30.2 (95% CI: 20.8 - NA, death events = 10) months (P = 0.21). Significant impacts on survival times were observed when comparing Young versus Old Females for patients with low (mOS of 21.3 versus 9.2 months, respectively; P = 0.001) and high (mOS of 30.2 versus 7 months, respectively; P = 0.004) levels of *TGFB2* methylation. [**C**] We examined the impact of *TGFB1* methylation on survival times, showing that the Old Male patients experienced similar trends in the shifts of the survival curves to *TGFB2* methylation: The mOS for 68 Old Male patients with low levels of *TGFB1* methylation exhibited mOS of 10.8 (95% CI: 7.6 - 14, death events = 56) months, and for 23 patients with high levels of *TGFB1* methylation experienced mOS of 12.2 (95% CI: 5.4 - NA, death events = 14) months (P = 0.46). Young Male patients showed improved survival times at high (N = 19; mOS = 21.1 (95% CI: 17.6 - NA, death events = 11) months) compared to low levels (N=61; mOS = 14 (95% CI: 11.8 - 16.6, death events = 44) months) of *TGFB1* methylation that was not statistically significant (P=0.18). At low levels of *TGFB1* methylation, Young Males experienced improved survival times compared to Old Males (14 versus 10.8 months, respectively; P = 0.049). [**D**] Old Females with low (N=47; mOS = 12.4 (95% CI: 8 - 15.8, death events = 37) months) and high (N=16; mOS = 7.4 (95% CI: 3.6 - NA, death events = 11) months) levels of *TGFB1* methylation experienced short mOS times that was not statistically significant (P = 0.84). Young Females with high levels of *TGFB1* methylation experienced the longest mOS time (N=15; mOS = 36.9 (95% CI: 18.6 - NA, death events = 6) months), but this was not statistically significant for patients with low levels of *TGFB1* methylation (N = 43; mOS = 21.3 (95% CI: 17.5 - 32.1, death events = 28) months; P = 0.24). Age significantly impacted mOS at low (Young versus Old Females mOS were 21.3 and 12.4 months, respectively; P < 0.0001) levels of *TGFB1* methylation. [**E**] Our examination of *MGMT* methylation, the mOS times for Males were: 10.8 (N=71; 95% CI: 7.1 - 14, death events = 57) months for Old *MGMT*^{lowMe} Males; 12.7 (N =20; 95% CI: 5.9 - NA, death events = 13) months for Old *MGMT*^{highMe} Males; 14 (N = 60; 95% CI: 11.8 - 18.1, death events = 41) months for Young *MGMT*^{lowMe} Males; and 17.8 (N = 20; 95% CI: 14 - NA, death events = 14) months for Young *MGMT*^{highMe} Males. The levels of *MGMT* methylation had no impact on Old Males (P = 0.37) or Young Males (P = 0.29). At low *MGMT* methylation, Young Males displayed longer mOS than Old Males (mOS of 14 versus 10.8 months, respectively; P = 0.044). [**F**] In Female patients, the following mOS times were observed: 9.1 (N = 47; 95% CI: 5.4 - 15.8, death events = 38) months for Old *MGMT*^{lowMe} Females; 12.4 (N = 16; 95% CI: 6.3 - NA, death events = 10) months for Old *MGMT*^{highMe} Females; 20.8 (N = 41; 95% CI: 15 - 33.2, death events = 26) months for Young *MGMT*^{lowMe} Females; 30 (N = 17; 95% CI: 21.3 - NA, death events = 8) months for Young *MGMT*^{highMe} Females. The levels of *MGMT* methylation had no impact on Old Females (P = 0.5) or Young Females (P = 0.13). At low levels of *MGMT* methylation, Young Females displayed longer mOS than Old Females (mOS of 20.8 versus 9.1 months, respectively; P = 0.001). At high levels of *MGMT* methylation, Young Females displayed longer mOS than Old Females (mOS of 30 versus 12.4 months, respectively; P = 0.003). [**G**] The impact of *TGFB3* methylation in Male patients, the mOS times were as follows: 12.6 (N = 71; 95% CI: 9.4 - 14.7, death events = 56) months for Old *TGFB3*^{lowMe} patients; 9.5 (N = 20; 95% CI: 3.7 - NA, death events = 14) months for Old *TGFB*^{highMe} patients; 14 (N = 59; 95% CI: 11.4 - 16.7, death events = 40) months for Young *TGFB3*^{lowMe} patients; and 18.1 (N = 21; 95% CI: 15 - NA, death events = 15) months for Young *TGFB3*^{highMe} patients. At high levels of *TGFB3* methylation, Young Males exhibited improved survival times compared to Old Males (mOS = 18.1 versus 9.5 months, respectively; P = 0.0119). [**H**] In Female patients the following mOS times were observed: 12.4 (N = 45; 95% CI: 8 - 16, death events = 32) months for Old *TGFB3*^{lowMe} patients; 7.2 (N = 18; 95% CI: 4.1 - 19, death events = 16) months for Old *TGFB3*^{highMe} patients; 20.8 (N = 44; 95% CI: 17.2 - 32.1, death events = 26) months for Young *TGFB3*^{lowMe} patients; and 36.9 (N = 14; 95% CI: 22.5 - NA, death events = 8) months for Young *TGFB3*^{highMe} patients. At high levels of *TGFB3* methylation, Young Females exhibited improved survival times compared to Old Females (mOS = 36.9 versus 7.2 months, respectively; P < 0.0001). At low levels of *TGFB3* methylation, Young Females exhibited improved survival times compared to Old Females (mOS = 20.8 versus 12.4 months, respectively; P = 0.005).

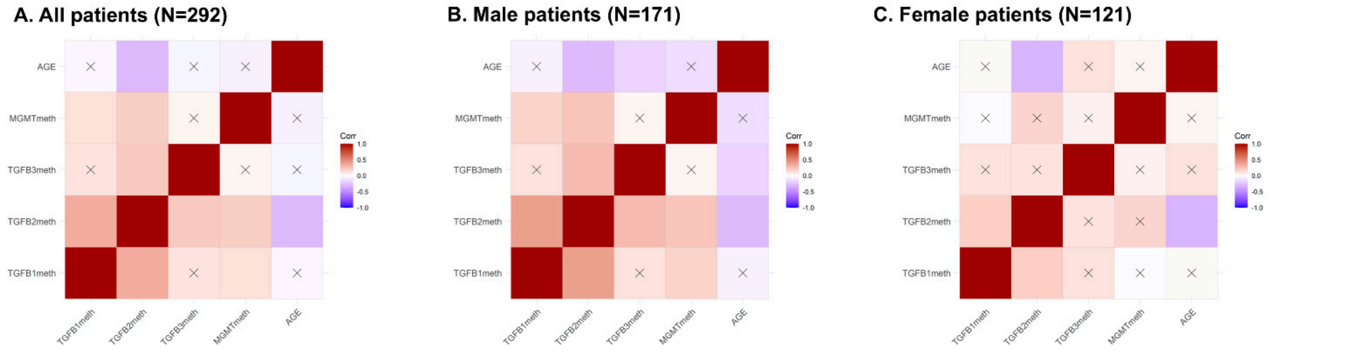

Variable 1	Variable 2	Correlation Coefficient	P-value
TGFB2meth	TGFB1meth	0.35	<1E-08
TGFB3meth	TGFB1meth	0.11	5.21E-02
MGMTmeth	TGFB1meth	0.13	3.12E-02
AGE	TGFB1meth	-0.03	6.47E-01
TGFB3meth	TGFB2meth	0.22	2.06E-04
MGMTmeth	TGFB2meth	0.21	2.80E-04
AGE	TGFB2meth	-0.3	2.00E-07
MGMTmeth	TGFB3meth	0.04	4.56E-01
AGE	TGFB3meth	-0.04	4.93E-01
AGE	MGMTmeth	-0.06	2.94E-01

Variable 1	Variable 2	Correlation Coefficient	P-value
TGFB2meth	TGFB1meth	0.4	1.00E-07
TGFB3meth	TGFB1meth	0.12	1.11E-01
MGMTmeth	TGFB1meth	0.18	1.71E-02
AGE	TGFB1meth	-0.05	4.97E-01
TGFB3meth	TGFB2meth	0.28	1.95E-04
MGMTmeth	TGFB2meth	0.24	1.59E-03
AGE	TGFB2meth	-0.29	1.04E-04
MGMTmeth	TGFB3meth	0.04	5.84E-01
AGE	TGFB3meth	-0.18	2.14E-02
AGE	MGMTmeth	-0.14	7.82E-02

Variable 1	Variable 2	Correlation Coefficient	P-value
TGFB2meth	TGFB1meth	0.2	2.78E-02
TGFB3meth	TGFB1meth	0.12	1.81E-01
MGMTmeth	TGFB1meth	-0.01	8.81E-01
AGE	TGFB1meth	0.02	8.04E-01
TGFB3meth	TGFB2meth	0.11	2.20E-01
MGMTmeth	TGFB2meth	0.17	6.39E-02
AGE	TGFB2meth	-0.32	3.35E-04
MGMTmeth	TGFB3meth	0.05	5.91E-01
AGE	TGFB3meth	0.12	2.04E-01
AGE	MGMTmeth	0.04	6.81E-01

Figure S3. Correlations of variables investigated in the multivariate Cox proportional hazards model.

Pearson r coefficients were determined for pairwise correlations for All (A), Male (B), and Female GBM patients from the TCGA dataset for *TGFB1/2/3* and *MGMT* beta values and age at diagnosis (ggcorrplot_0.1.4.1implemented in R). The cluster figure depicts a matrix of correlation coefficients (corr) for each pairwise correlation ranging from positive (dark red) to negative r values (blue). Cells with "X" indicate non-significant correlations at P>0.05. [**A**] In all GBM patients, *TGFB2* methylation was positively correlated with *TGFB1* (r = 0.35, P<0.0001), *TGFB3* (r = 0.22, P = 0.0002), and *MGMT* (r = 0.21, P=0.0003) methylation and negatively correlated to the age at diagnosis (r = -0.3, P<0.0001). [**B**] Correlation of variables in Male patients showed *TGFB2* methylation was positively correlated with *TGFB1* (r = 0.4, P<0.0001), *TGFB3* (r = 0.28, P = 0.0002) and *MGMT* (r = 0.24, P=0.0016) methylation and negatively correlated to the age at diagnosis (r = -0.29, P=0.0001). [**C**] Female patients exhibited weaker correlation than male patients whereby *TGFB2* methylation was only correlated to *TGFB1* methylation (r = 0.2, P=0.028) and age at diagnosis (r = -0.32, P = 0.0003).

A

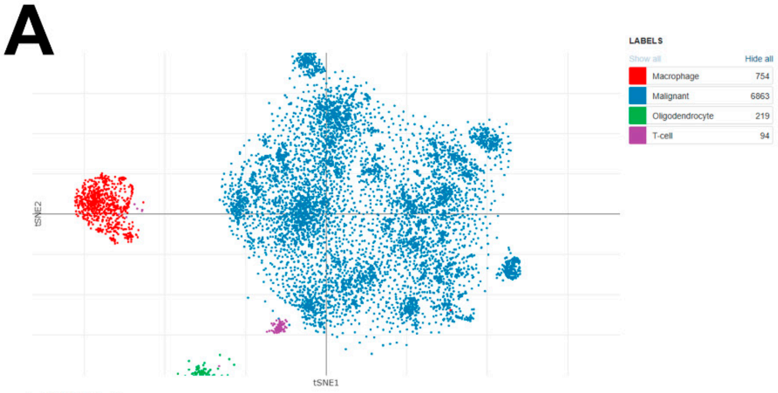

	% Cells				Log2 TPM			
	Macrophage	Malignant	Oligodendrocyte	T-cell	Macrophage	Malignant	Oligodendrocyte	T-cell
PARP9	50.8	43.67	42.92	41.49	1.45	1.16	1.26	1.1
TRIM21	18.7	11.88	9.13	11.7	0.56	0.34	0.28	0.42
IFI35	24.14	15.27	14.61	13.83	0.84	0.47	0.6	0.52
TRIM22	80.5	26.52	5.02	78.72	3.22	0.81	0.16	3.96
IFIH1	18.17	12.24	18.72	11.7	0.38	0.28	0.47	0.34
STAT1	44.16	53.3	62.56	36.17	1.58	1.88	2.82	1.56
TRIM5	46.02	46.54	31.51	44.68	0.61	0.69	0.3	0.32
HIF1A	89.26	81.82	47.03	51.06	4.82	3.55	1.41	1.85
RSAD2	18.7	19.41	17.81	28.72	0.21	0.18	0.33	0.44
IFIT3	67.37	68.64	74.89	70.21	0.8	0.84	2.3	0.61
IRAK4	92.18	90.05	86.3	91.49	1.16	1.03	0.69	1.26
BCL10	43.37	43.03	23.74	20.21	1.04	1	0.47	0.48
CAPZA1	94.83	82.86	76.71	88.3	4.78	3.3	2.94	4.79
PSMA4	51.99	72.52	64.84	31.91	2.36	3.5	3.04	1.61
NUDT12	3.58	13.13	20.55	2.13	0.1	0.35	0.71	0.05
SNAP23	66.31	46.34	47.95	36.17	2.4	1.13	1.38	1.06
UBA3	35.94	59.16	63.47	22.34	1.37	2.35	2.89	0.93
ITCH	49.6	66.24	85.39	45.74	0.92	1.35	2.16	1.03
MALT1	30.5	12.21	2.74	25.53	0.52	0.2	0.04	0.5
HLA-DRB1	98.01	3.32	0.46	43.62	7.6	0.11	0.02	2
CD86	80.77	0.35	0	3.19	3.46	0.01	0	0.12
CD3D	0.53	0.07	0	84.04	0.02	0	0	5.8
CD3E	0.53	0.1	0	87.23	0.01	0	0	4.32

B

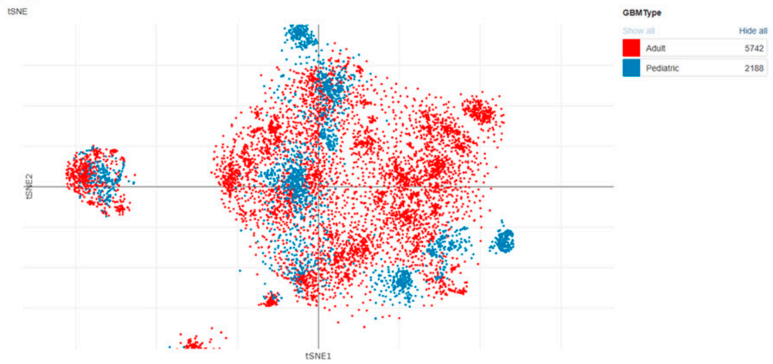

	% Cells		Log2 TPM	
Gene	Adult	Pediatric	Adult	Pediatric
PARP9	47.14	36.84	1.33	0.81
TRIM21	12.47	12.39	0.36	0.36
IFI35	17.49	12.39	0.56	0.38
TRIM22	36.17	19.88	1.22	0.63
IFIH1	14.8	8.18	0.34	0.17
STAT1	53.34	50.23	1.97	1.62
TRIM5	46.95	43.69	0.73	0.5
HIF1A	81.23	81.12	3.72	3.26
RSAD2	19.61	18.88	0.22	0.1
IFIT3	68.55	69.15	0.92	0.75
IRAK4	89.74	91.27	1.07	0.94
BCL10	40.67	46.44	0.94	1.08
CAPZA1	82.88	86.56	3.38	3.65
PSMA4	66.6	78.47	3.2	3.78
NUDT12	12.14	12.71	0.34	0.32
SNAP23	46.88	51.51	1.24	1.32
UBA3	54.72	61.65	2.22	2.36
ITCH	66.06	62.02	1.38	1.21
MALT1	13.81	13.94	0.24	0.21
HLA-DRB1	13.01	11.97	0.84	0.87
CD86	8.03	8	0.34	0.32
CD3D	1.36	0.46	0.09	0.03
CD3E	1.46	0.41	0.07	0.02

Figure S4. Clustering using a tSNE projection of cells isolated from wild-type GBM patients.
We analyzed the expression of the 23 genes in an independent dataset utilizing RNA sequencing quantification from single cells obtained from 20 adult and 8 pediatric *IDH*-wildtype glioblastoma patients. The figure shows color coded separation of cell types using the tSNE algorithm accompanied with table depicting the percentage composition and Log2 TPM expression levels of each of the 23 genes for: (**A**). Macrophage (754 cells), Malignant (6863 cells), Oligodendrocyte (219 cells), and T-cells (94 cells) and (**B**) GBM type (Adult: 5742 cells, and Pediatric: 2188, cells).

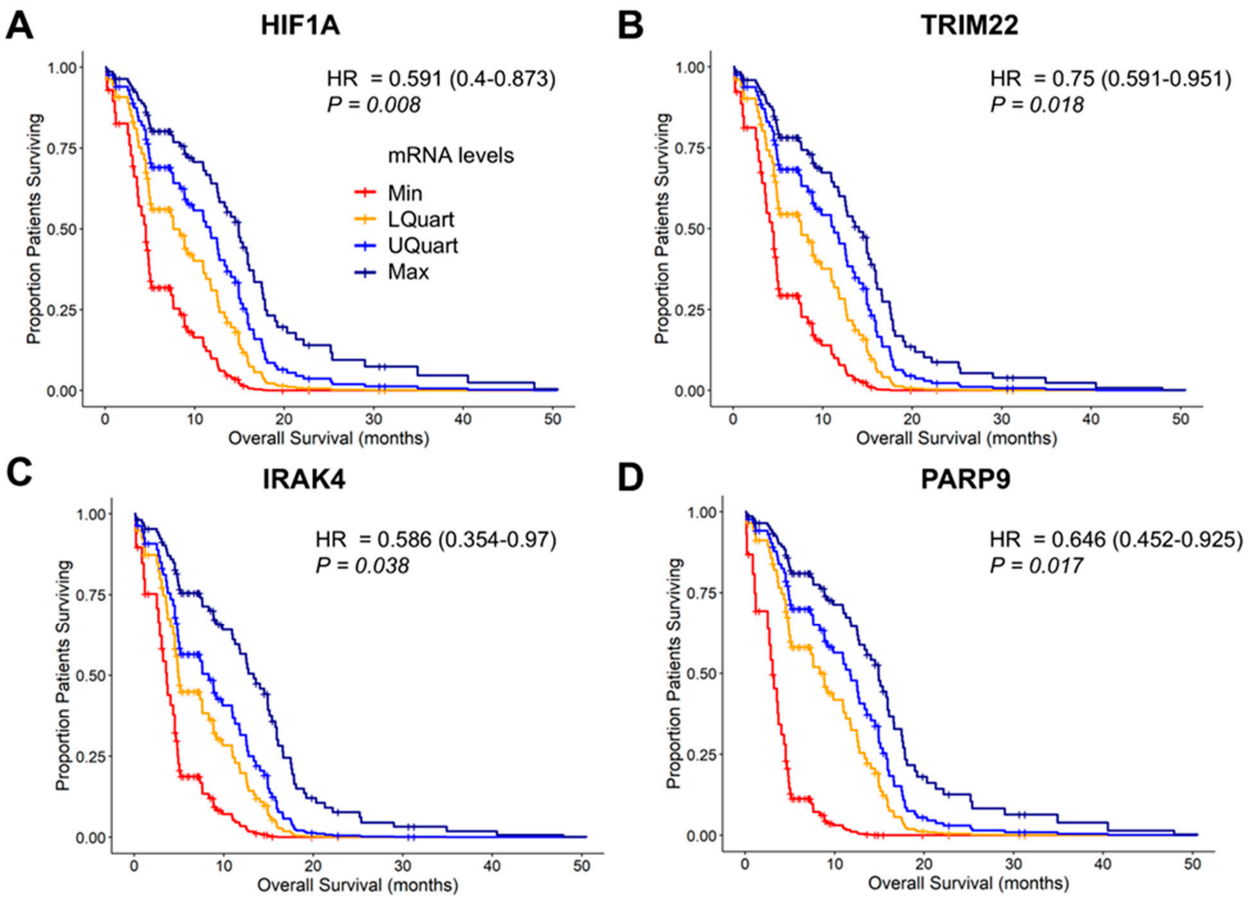

Figure S5. Prognostic impact of genes highly expressed in adult macrophages identified from bulk and single-cell RNA-seq experiments.

Four genes exhibited a high level of expression in more than 50% of the macrophages and were further investigated for their positive prognostic impact on OS in GBM patients (HIF1A: 89.26% of macrophage cells, 4.82 Log2 TPM; TRIM22: 80.5%, 3.22 Log2 TPM; IRAK4 92.18%, 1.16 Log2 TPM; PARP9: 50.8%, 1.45 Log2 TPM; compared to the M1-like macrophage positive control CD86: 80.77%, 3.46Log2 TPM). In multivariate models, increasing age and male patients exhibited worse survival outcomes, prompting us to investigate the favorable prognostic impact of genes identified in the TCGA and single-cell RNA seq experiments. The fitted parameters from the multivariate Cox proportional hazards model were used to generate survival curves for increasing levels of A. HIF1A, B. TRIM22,C. IRAK4, and D. PARP9 mRNA (ranging from minimum (Min); lower quartile (LQuart); upper quartile (UQuart); and maximum (Max) levels of mRNA expression) for Old Males (89 yr) of GBM patients OS was followed with the gene methylation variables set to low levels of *TGFB1/3* and *MGMT.* [**A**] Increasing mRNA levels of HIF1A resulted in progressively more favorable survival outcomes: The expression levels of 10.668, 11.964, 12.812, and 13.8 log2 (TPM) HIF1A corresponded to median survivals of 4.5, 7.6, 11.7, and 14.9 months, respectively. [**B**] For TRIM22, the increasing expression levels of 7.208, 9.651, 11.275, and 12.794 log2 (TPM) corresponded to median survivals of 4.4, 7.6, 11.3, and 14 months, respectively. [**C**] For IRAK4, the expression levels of 7.244, 8.621, 9.254, and 10.578 log2 (TPM) corresponded to median survivals of 3.6, 4.9, 8.4, and 13.3 months, respectively. [**D**] For PARP9, the increasing expression levels of 7.036, 10.212, 11.173, and 12.377 log2 (TPM) corresponded to median survivals of 3.1, 8.4, 11.7, and 14.9 months, respectively.

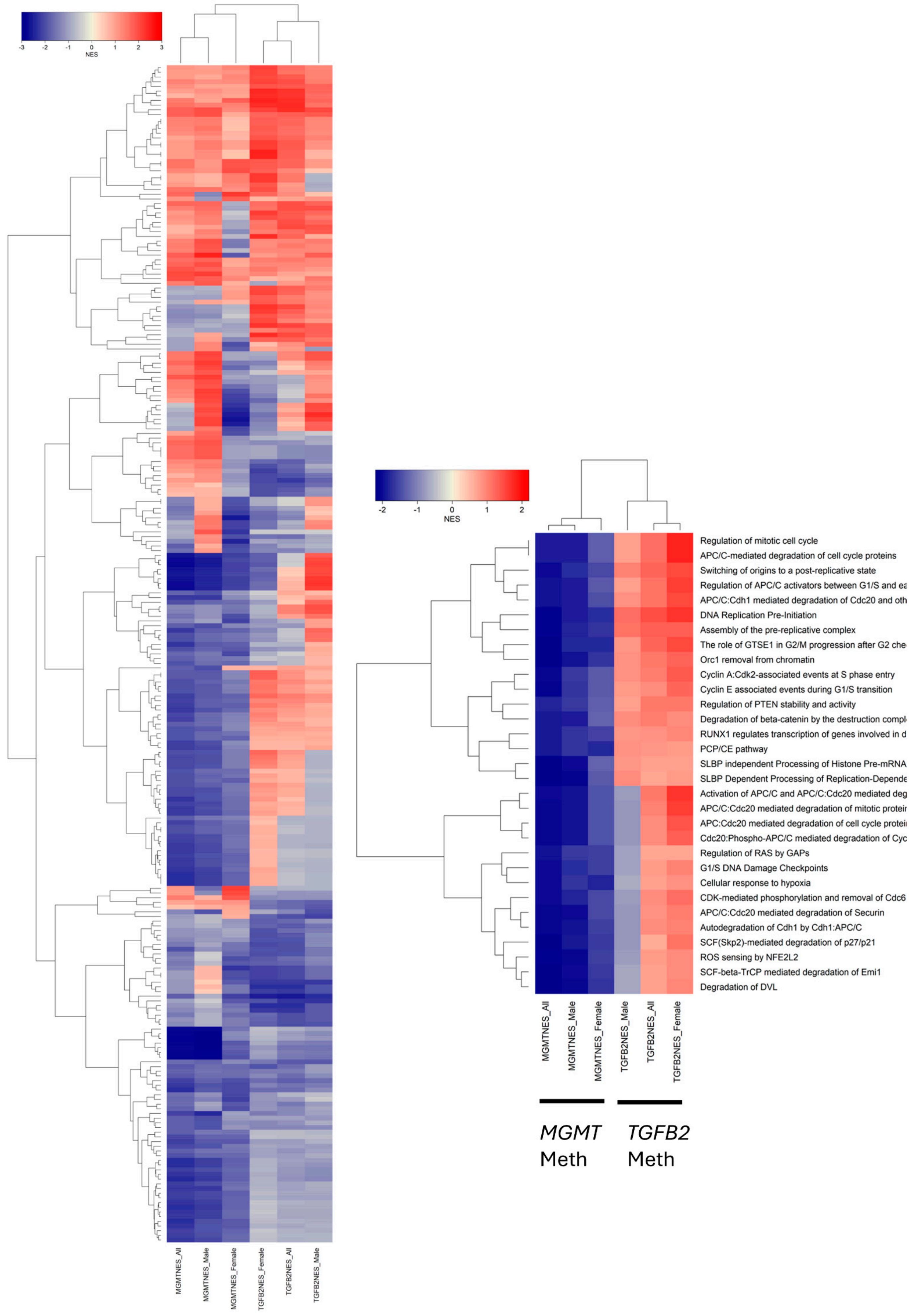

Figure S6. Identification of Reactome pathways negatively correlated to *MGMT* methylation compared to *TGFB2* methylation.

Beta-values for *TGFB2* and *MGMT* methylation were correlated with mRNA expression levels of 14364 genes for all GBM patients and subsets stratified according to sex (Male (N=59) and Female (N=42)). The cluster figure depicts the NES scores for *MGMT* methylation for All (MGMTNES_All), Female (MGMTNES_Female), and Male patients (MGMT_Male), and for *TGFB2* methylation (TGFB2NES_All, TGFB2NES_Female, TGFB2NES_Male). The multivariate Cox regression curves exhibited sexual dimorphism in the OS response comparing young males and young females with high levels of *MGMT* methylation, which was reduced for high levels of *TGFB2* methylation. We identified 31 Reactome pathways that exhibited NES scores enriched with significant negative correlated mRNA expression of genes with *MGMT* methylation in males and females (P<0.001 for NES scores) and showed positive or reduced negative NEScores for *TGFB2* methylation. These 31 pathways were represented by 327 genes, of which 45 exhibited a significant negative correlation with MGMT methylation (P<0.05).

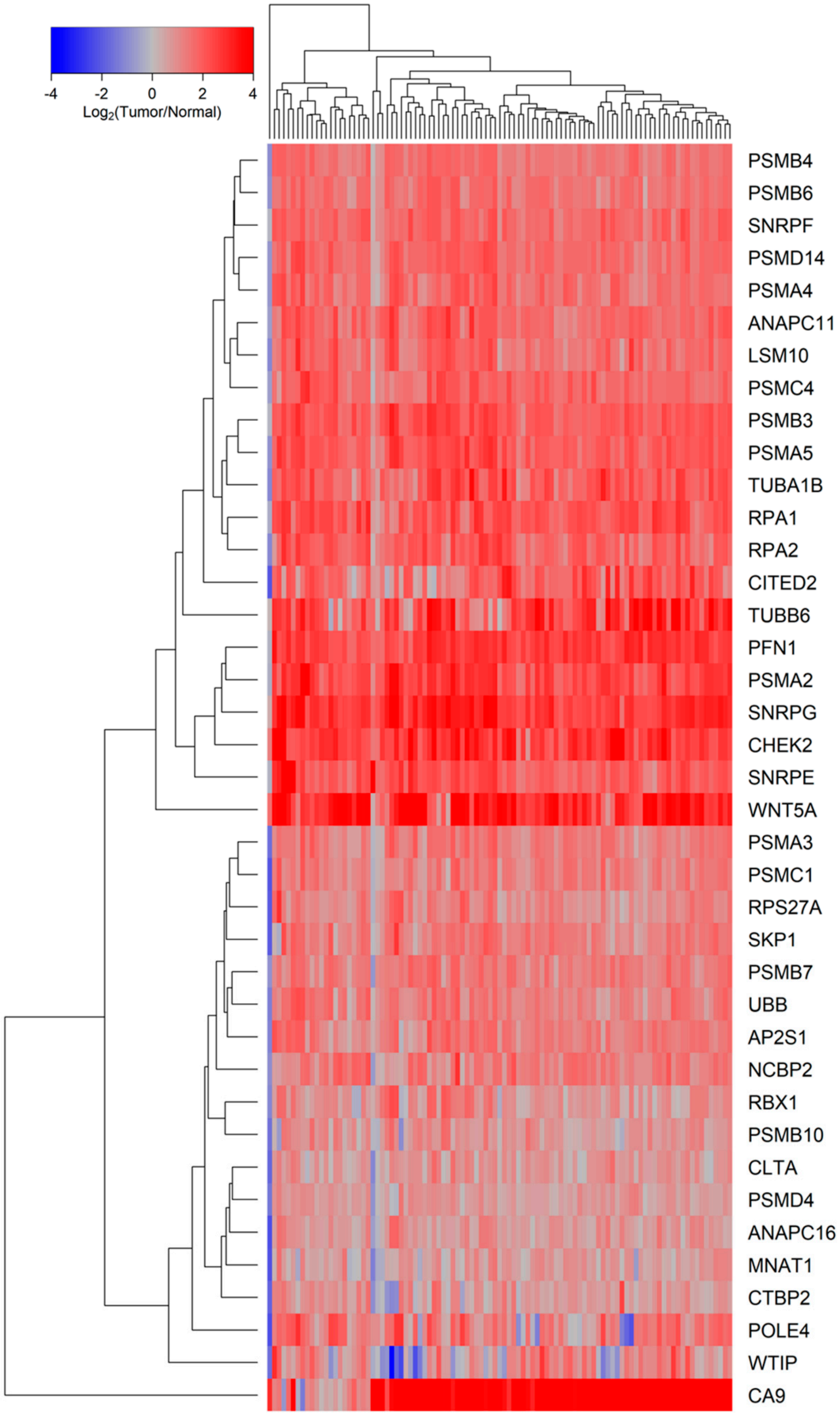

Figure S7. Upregulation of genes correlated with *MGMT* methylation.

Out of the 45 genes negatively correlated with MGMT methylation, 39 exhibited significant upregulation in tumor versus normal tissue (P<0.0001).

Table S1. Reactome Pathways negatively correlated with *TGFB2* Methylation.

Cluster Index	Reactome Pathway	Normalized Enrichment Score		
		Old	All	Young
1	MyD88:MAL(TIRAP) cascade initiated on plasma membrane	-1.77	-1.25	-1.08
2	Toll Like Receptor TLR6:TLR2 Cascade	-1.77	-1.25	-1.08
3	Toll Like Receptor 2 (TLR2) Cascade	-1.79	-1.24	-1.07
4	Toll Like Receptor TLR1:TLR2 Cascade	-1.79	-1.24	-1.07
5	Toll Like Receptor 3 (TLR3) Cascade	-1.78	-1.21	-1.13
6	MyD88-independent TLR4 cascade	-1.84	-1.27	-1.13
7	TRIF(TICAM1)-mediated TLR4 signaling	-1.84	-1.27	-1.13
8	CLEC7A (Dectin-1) signaling	-1.74	-1.43	-1.17
9	Interleukin-12 family signaling	-1.98	-1.48	-1.11
10	Interleukin-4 and Interleukin-13 signaling	-2.00	-1.35	-1.04
11	Chemokine receptors bind chemokines	-1.93	-1.62	-1.32
12	RHOH GTPase cycle	-1.90	-1.60	-1.28
13	COPII-mediated vesicle transport	-1.86	-1.57	-1.34
14	ER-Phagosome pathway	-1.86	-1.59	-1.31
15	TNFR2 non-canonical NF-kB pathway	-1.78	-1.63	-1.29
16	Fcgamma receptor (FCGR) dependent phagocytosis	-2.18	-1.43	-1.00
17	Role of LAT2/NTAL/LAB on calcium mobilization	-2.14	-1.44	-1.03
18	RHO GTPases Activate WASPs and WAVEs	-2.25	-1.51	-1.01
19	RHO GTPases Activate NADPH Oxidases	-2.20	-1.66	-1.13
20	FCGR3A-mediated phagocytosis	-2.10	-1.25	-0.89
21	Leishmania phagocytosis	-2.10	-1.25	-0.89
22	Parasite infection	-2.10	-1.25	-0.89
23	Regulation of actin dynamics for phagocytic cup formation	-2.06	-1.27	-0.85
24	EPHB-mediated forward signaling	-1.99	-1.27	-0.88
25	Toll Like Receptor 7/8 (TLR7/8) Cascade	-1.78	-1.14	-0.95
26	Toll Like Receptor 9 (TLR9) Cascade	-1.80	-1.11	-0.94
27	MyD88 dependent cascade initiated on endosome	-1.73	-1.09	-0.96
28	Interleukin receptor SHC signaling	-2.21	-1.84	-1.29
29	Nucleotide-binding domain, leucine rich repeat containing receptor (NLR) signaling pathways	-2.18	-1.70	-1.27
30	Cargo concentration in the ER	-2.04	-1.75	-1.35
31	Interleukin-3, Interleukin-5 and GM-CSF signaling	-2.06	-1.83	-1.23
32	Downstream TCR signaling	-2.04	-1.60	-1.33
33	GPVI-mediated activation cascade	-2.07	-1.60	-1.22
34	NOD1/2 Signaling Pathway	-1.95	-1.70	-1.22
35	CD28 dependent Vav1 pathway	-2.18	-1.79	-1.56
36	Antigen processing-Cross presentation	-2.19	-1.82	-1.51
37	Dectin-2 family	-2.08	-1.62	-1.48
38	Costimulation by the CD28 family	-2.50	-1.88	-1.44
39	Cell recruitment (pro-inflammatory response)	-2.01	-1.84	-1.67
40	Purinergic signaling in leishmaniasis infection	-2.01	-1.84	-1.67
41	TNFs bind their physiological receptors	-1.98	-2.00	-1.66
42	IRAK4 deficiency (TLR2/4)	-2.03	-2.02	-1.83

43	MyD88 deficiency (TLR2/4)	-1.92	-1.97	-1.78
44	Diseases associated with the TLR signaling cascade	-2.19	-2.07	-1.75
45	Diseases of Immune System	-2.19	-2.07	-1.75
46	Interleukin-10 signaling	-2.11	-2.07	-1.78
47	Interleukin-2 family signaling	-2.36	-2.21	-1.74
48	Regulation of Complement cascade	-1.50	-1.96	-1.82
49	Complement cascade	-1.44	-1.97	-1.82
50	Nicotinate metabolism	-1.50	-2.00	-1.76
51	Regulation of TLR by endogenous ligand	-1.63	-2.01	-1.81
52	Antigen activates B Cell Receptor (BCR) leading to generation of second messengers	-1.65	-1.90	-1.74
53	Acyl chain remodelling of PC	-1.49	-1.82	-1.97
54	NF-kB activation through FADD/RIP-1 pathway mediated by caspase-8 and -10	-1.26	-2.02	-2.00
55	Metabolic disorders of biological oxidation enzymes	-0.93	-1.94	-1.89
56	Phase II - Conjugation of compounds	-1.08	-1.89	-1.74
57	Mitochondrial translation	-0.72	-1.54	-1.80
58	PD-1 signaling	-2.38	-2.36	-2.02
59	Phosphorylation of CD3 and TCR zeta chains	-2.39	-2.34	-2.02
60	Translocation of ZAP-70 to Immunological synapse	-2.41	-2.29	-2.05
61	Generation of second messenger molecules	-2.41	-2.26	-2.07
62	Interferon gamma signaling	-2.37	-2.30	-2.37
63	Interferon alpha/beta signaling	-2.50	-2.26	-2.23
64	Immunoregulatory interactions between a Lymphoid and a non-Lymphoid cell	-2.39	-2.50	-2.43

Table S2. Tumor versus Normal mRNA Expression of Genes correlated with *TGFB2* methylation.

Gene	Normal Expression (Mean Log2 TPM ± SEM)	Tumor Expression (Mean Log2 TPM ± SEM)	Fold change (Tumor/Normal)	P-value
ABHD14B	3.48 ± 0.04	4.66 ± 0.06	2.27	2.18E-11
ACTR2	5.26 ± 0.04	6.3 ± 0.06	2.06	3.98E-09
ACY1	3.07 ± 0.03	4.62 ± 0.06	2.94	1.67E-18
ALOX5	2.21 ± 0.07	3.55 ± 0.11	2.53	4.03E-14
ANXA1	3.35 ± 0.05	8.02 ± 0.14	25.38	8.17E-153
ARHGDIB	3.78 ± 0.04	7.22 ± 0.08	10.85	5.43E-84
ARPC1B	3.77 ± 0.04	6.61 ± 0.09	7.15	8.77E-58
ARPC2	6.29 ± 0.04	7.75 ± 0.05	2.75	1.53E-16
ARPC3	6.58 ± 0.04	8.16 ± 0.05	3.00	3.67E-19
ARPC4	5.52 ± 0.04	7.55 ± 0.05	4.09	1.85E-30
BATF	-2.21 ± 0.1	0.75 ± 0.12	7.79	1.01E-62
BCL10	0.87 ± 0.03	2.77 ± 0.05	3.73	8.82E-27
BCL2	1.49 ± 0.03	1.82 ± 0.08	1.26	6.40E-02
BET1	2.84 ± 0.03	4.6 ± 0.08	3.39	2.54E-23
BLNK	-0.04 ± 0.06	2.25 ± 0.12	4.90	2.29E-38
BST2	4.22 ± 0.04	6.89 ± 0.14	6.34	4.19E-51
BTK	0.16 ± 0.05	2.75 ± 0.1	6.00	2.76E-48
C1S	3.61 ± 0.04	6.23 ± 0.14	6.16	1.27E-49
C3	5.18 ± 0.05	9.28 ± 0.13	17.21	8.46E-119
C3AR1	0.3 ± 0.06	4.39 ± 0.11	17.13	2.14E-118
CAPZA1	4.21 ± 0.03	5.8 ± 0.05	3.01	2.94E-19
CARD9	0.91 ± 0.04	1.71 ± 0.09	1.74	6.00E-06
CASP1	1.36 ± 0.04	4.62 ± 0.11	9.58	1.31E-75
CASP10	0.19 ± 0.03	0.99 ± 0.09	1.74	6.25E-06
CASP4	1.58 ± 0.04	4.73 ± 0.09	8.89	7.49E-71
CASP8	0.24 ± 0.03	2.78 ± 0.08	5.83	8.64E-47
CCL2	1.91 ± 0.07	6.36 ± 0.17	21.90	2.20E-139
CCR1	-1.06 ± 0.06	2.94 ± 0.11	15.95	1.20E-112
CCR2	-5.4 ± 0.08	-1.35 ± 0.22	16.58	9.32E-116
CCR5	-3.34 ± 0.07	0.9 ± 0.13	18.88	1.66E-126
CCR6	-3.66 ± 0.09	-2.88 ± 0.17	1.71	1.24E-05
CCRL2	-1.28 ± 0.04	1.63 ± 0.08	7.55	6.64E-61
CD1C	-8.44 ± 0.08	-3.14 ± 0.28	39.46	1.05E-196
CD1D	-3.45 ± 0.06	-0.03 ± 0.12	10.72	3.81E-83
CD226	-2.13 ± 0.04	-0.7 ± 0.11	2.69	6.71E-16
CD274	-0.4 ± 0.04	1.1 ± 0.13	2.83	2.12E-17
CD300C	-3.25 ± 0.08	1.34 ± 0.12	24.03	9.19E-148
CD300LB	-5.91 ± 0.08	-1.77 ± 0.11	17.69	4.56E-121
CD300LF	-1.65 ± 0.06	1.31 ± 0.12	7.73	2.57E-62
CD33	-0.05 ± 0.06	3.08 ± 0.1	8.75	7.37E-70
CD3D	-4.14 ± 0.11	0.45 ± 0.21	24.10	4.96E-148
CD3E	-2.53 ± 0.07	0.58 ± 0.18	8.63	5.23E-69
CD40	2.08 ± 0.04	2.79 ± 0.09	1.64	5.61E-05
CD46	4.45 ± 0.04	4.73 ± 0.06	1.21	1.23E-01
CD79B	-1.73 ± 0.04	-0.13 ± 0.15	3.04	1.22E-19
CD80	-6.52 ± 0.09	-2.05 ± 0.12	22.09	3.88E-140
CD86	-0.74 ± 0.06	3.31 ± 0.11	16.49	2.52E-115
CD96	-4.07 ± 0.07	-1.57 ± 0.15	5.64	4.09E-45
CDC42	5.87 ± 0.04	7.69 ± 0.05	3.54	8.04E-25
CFB	2.52 ± 0.04	3.4 ± 0.16	1.83	8.01E-07
CFH	2.24 ± 0.04	3.6 ± 0.14	2.56	1.92E-14
CHAC1	1.79 ± 0.05	1.41 ± 0.11	0.77	3.29E-02
CHUK	2.46 ± 0.03	3.01 ± 0.06	1.47	1.83E-03
CLEC4A	0.58 ± 0.03	1.98 ± 0.09	2.64	2.69E-15
CLEC7A	0.42 ± 0.06	2.88 ± 0.11	5.51	6.63E-44
COL17A1	-0.8 ± 0.06	-3.37 ± 0.2	0.17	1.23E-47
COLEC11	-0.19 ± 0.05	0.2 ± 0.17	1.31	2.77E-02
CRTAM	-1.28 ± 0.13	-2.28 ± 0.21	0.50	1.43E-08
CSF2RA	0.56 ± 0.05	3.59 ± 0.11	8.18	1.06E-65
CTLA4	-4.19 ± 0.08	-2.39 ± 0.25	3.48	3.11E-24
CTSS	1.44 ± 0.05	5.57 ± 0.12	17.49	4.33E-120

CX3CR1	0.6 ± 0.06	4.35 ± 0.15	13.45	1.87E-99
CXCL9	-5.01 ± 0.09	0.4 ± 0.17	42.60	6.67E-205
CYBA	3.99 ± 0.05	6.9 ± 0.09	7.49	1.94E-60
CYBB	0.57 ± 0.05	4.25 ± 0.13	12.86	4.10E-96
DAPP1	-2.96 ± 0.06	0.02 ± 0.12	7.85	3.07E-63
DBT	1.2 ± 0.03	1.87 ± 0.06	1.58	1.80E-04
DDX58	0.92 ± 0.03	2.58 ± 0.1	3.16	7.43E-21
DUSP3	4.77 ± 0.04	4.7 ± 0.06	0.95	6.99E-01
EDA2R	-1.84 ± 0.05	1.44 ± 0.15	9.70	1.94E-76
ENTPD1	2.2 ± 0.03	4.18 ± 0.08	3.94	5.58E-29
ESD	5.43 ± 0.04	6.73 ± 0.06	2.46	2.27E-13
F8	2.36 ± 0.03	3.31 ± 0.08	1.93	8.42E-08
FCER1G	3.48 ± 0.06	7.4 ± 0.1	15.18	9.72E-109
FCGR1A	2.15 ± 0.06	5.9 ± 0.11	13.45	1.99E-99
FCGR3A	2.23 ± 0.06	6.69 ± 0.12	22.06	4.74E-140
FOLR1	-0.84 ± 0.06	2.11 ± 0.19	7.73	2.80E-62
GATA3	-4.27 ± 0.07	-1.71 ± 0.2	5.89	2.78E-47
GBP3	0.87 ± 0.04	3.69 ± 0.19	7.02	9.16E-57
GBP5	-2.57 ± 0.06	0.95 ± 0.14	11.48	6.72E-88
GBP6	-4.75 ± 0.06	-3.31 ± 0.22	2.71	5.14E-16
GCLM	1.83 ± 0.03	3.41 ± 0.08	2.98	5.91E-19
GLYATL2	-2.13 ± 0.06	-1.4 ± 0.21	1.66	3.92E-05
GSDMD	3.13 ± 0.03	4.84 ± 0.11	3.29	3.18E-22
GSTK1	5.11 ± 0.04	7.29 ± 0.07	4.52	9.93E-35
GSTM4	4.65 ± 0.04	5.47 ± 0.07	1.76	3.74E-06
HAVCR2	1.07 ± 0.04	4.24 ± 0.1	9.04	6.94E-72
HCST	2.02 ± 0.05	4.76 ± 0.1	6.64	1.11E-53
HIF1A	4.2 ± 0.04	6.63 ± 0.08	5.37	1.15E-42
HLA-DPA1	3.82 ± 0.05	7.81 ± 0.12	15.94	1.38E-112
HLA-DPB1	4.09 ± 0.05	7.41 ± 0.11	10.03	1.22E-78
HLA-DQA1	-1.86 ± 0.08	3.36 ± 0.19	37.25	0.00E+00
HLA-DQB1	1.86 ± 0.06	5.42 ± 0.19	11.82	5.40E-90
HLA-DRA	4.59 ± 0.06	9.46 ± 0.12	29.35	1.16E-166
HLA-DRB1	3.52 ± 0.05	7.71 ± 0.12	18.22	1.69E-123
HPGDS	-1.37 ± 0.07	2.39 ± 0.12	13.63	2.02E-100
HSPA8	8.63 ± 0.05	9.89 ± 0.06	2.39	1.22E-12
IFI27	6.11 ± 0.04	7.18 ± 0.14	2.10	1.62E-09
IFI35	2.76 ± 0.04	5.48 ± 0.1	6.58	3.90E-53
IFIH1	0.53 ± 0.03	2.87 ± 0.12	5.05	9.00E-40
IFIT1	4.23 ± 0.04	4.82 ± 0.15	1.51	7.95E-04
IFIT3	2.98 ± 0.04	4.79 ± 0.12	3.50	1.77E-24
IFNAR1	2.76 ± 0.03	4.31 ± 0.05	2.94	1.63E-18
IFNGR1	4.33 ± 0.04	6.14 ± 0.07	3.50	2.00E-24
IKBKB	3.9 ± 0.04	4.77 ± 0.06	1.83	9.43E-07
IKBKE	0.23 ± 0.04	2.07 ± 0.08	3.60	1.74E-25
IL12RB1	-1.99 ± 0.06	0.55 ± 0.1	5.82	1.21E-46
IL13RA1	2.16 ± 0.04	4.73 ± 0.09	5.95	8.70E-48
IL15	-0.63 ± 0.04	0.52 ± 0.12	2.22	8.77E-11
IL18	0.77 ± 0.05	4.12 ± 0.1	10.18	1.20E-79
IL1A	-4.08 ± 0.08	-0.58 ± 0.17	11.34	4.96E-87
IL2RB	-3.26 ± 0.05	-0.64 ± 0.15	6.16	1.35E-49
IL2RG	-0.11 ± 0.04	1.86 ± 0.11	3.92	8.17E-29
INPP5D	2.29 ± 0.04	3.77 ± 0.11	2.80	5.02E-17
IRAK3	-0.34 ± 0.04	0.49 ± 0.1	1.78	2.66E-06
IRAK4	1.2 ± 0.03	3.1 ± 0.07	3.73	7.77E-27
IRF5	1.1 ± 0.04	2.98 ± 0.09	3.68	2.73E-26
IRF8	0.32 ± 0.05	2.8 ± 0.11	5.56	2.31E-44
ITCH	2.79 ± 0.03	3.86 ± 0.06	2.11	1.24E-09
ITGA4	-1.34 ± 0.05	1.37 ± 0.12	6.54	7.90E-53
ITGAL	-0.43 ± 0.04	1.9 ± 0.11	5.02	1.97E-39
ITGAV	3.61 ± 0.04	5.66 ± 0.07	4.14	6.08E-31
ITGB7	-0.62 ± 0.04	0.15 ± 0.1	1.71	1.37E-05
JAK2	1.36 ± 0.04	1.91 ± 0.07	1.46	1.97E-03
JAK3	1.12 ± 0.04	0.82 ± 0.13	0.82	9.84E-02
KLRB1	-4.55 ± 0.09	-1.51 ± 0.17	8.23	5.02E-66
KLRD1	-2.11 ± 0.04	-1.85 ± 0.13	1.20	1.39E-01
LAIR1	1.37 ± 0.06	4.71 ± 0.11	10.14	2.03E-79

LAT2	1.99 ± 0.04	4.6 ± 0.09	6.11	3.77E-49
LCP1	1.38 ± 0.05	4.16 ± 0.1	6.86	1.92E-55
LCP2	1.62 ± 0.05	4.17 ± 0.09	5.86	4.79E-47
LILRA1	-1.77 ± 0.05	0.82 ± 0.12	6.02	2.04E-48
LILRA5	-2.94 ± 0.09	-0.35 ± 0.16	6.04	1.29E-48
LILRB1	0.32 ± 0.05	2.43 ± 0.12	4.31	1.10E-32
LMAN1	2.85 ± 0.04	4.42 ± 0.06	2.96	1.04E-18
LNPEP	2.24 ± 0.04	2.24 ± 0.07	1.00	9.85E-01
LPCAT2	0.61 ± 0.05	2.81 ± 0.09	4.58	2.57E-35
LY96	1.19 ± 0.05	4.73 ± 0.12	11.61	1.06E-88
LYN	1.25 ± 0.05	3.98 ± 0.09	6.67	6.34E-54
MALT1	1.3 ± 0.04	2.42 ± 0.07	2.17	2.59E-10
MAPK9	4.72 ± 0.05	4.16 ± 0.06	0.68	1.56E-03
MAT2A	6.52 ± 0.04	7.37 ± 0.07	1.81	1.32E-06
MCFD2	4.44 ± 0.04	5.54 ± 0.06	2.14	5.80E-10
MEF2A	3.57 ± 0.04	4.07 ± 0.07	1.41	5.44E-03
MEF2C	3.14 ± 0.05	4.01 ± 0.07	1.83	7.69E-07
MGST2	3.64 ± 0.03	5.36 ± 0.08	3.29	2.89E-22
MRPL27	5.52 ± 0.04	6.53 ± 0.05	2.01	1.31E-08
MRPL35	3.65 ± 0.03	4.86 ± 0.06	2.30	1.14E-11
MRPS33	4.96 ± 0.04	6.82 ± 0.07	3.64	7.10E-26
MRPS9	3.75 ± 0.03	4.21 ± 0.05	1.37	9.85E-03
MRRF	3.41 ± 0.04	3.93 ± 0.06	1.43	3.36E-03
MTIF2	3.04 ± 0.03	4.04 ± 0.06	2.00	1.77E-08
MTIF3	5.28 ± 0.03	5.42 ± 0.06	1.10	4.37E-01
MX2	0.38 ± 0.04	2.35 ± 0.15	3.93	6.61E-29
MYD88	1.92 ± 0.04	4.55 ± 0.08	6.19	7.42E-50
NAT1	-1.98 ± 0.04	1.35 ± 0.09	10.04	1.04E-78
NCF4	0.5 ± 0.05	3.24 ± 0.09	6.66	8.78E-54
NCKAP1L	-0.06 ± 0.05	3.36 ± 0.11	10.68	6.49E-83
NLRC4	-1.42 ± 0.04	0.27 ± 0.09	3.22	1.47E-21
NLRP1	6.59 ± 0.03	7.48 ± 0.09	1.85	4.87E-07
NMNAT1	1.19 ± 0.03	2.71 ± 0.06	2.86	1.06E-17
NMNAT3	2.21 ± 0.03	3.49 ± 0.13	2.44	3.85E-13
NOD2	-2.26 ± 0.05	-0.24 ± 0.11	4.07	2.90E-30
NUDT12	1.86 ± 0.03	3.3 ± 0.07	2.72	3.72E-16
OAS1	1.35 ± 0.04	4.8 ± 0.15	10.93	1.77E-84
OAS2	0.7 ± 0.04	3.41 ± 0.15	6.54	7.82E-53
OAS3	0.97 ± 0.04	3.76 ± 0.13	6.92	6.35E-56
OASL	-1.71 ± 0.05	1.73 ± 0.16	10.90	2.50E-84
P2RX4	2.92 ± 0.05	4.36 ± 0.06	2.70	5.40E-16
PAK1	4.84 ± 0.06	4.54 ± 0.08	0.81	8.85E-02
PARP14	1.51 ± 0.04	3.27 ± 0.11	3.38	3.43E-23
PARP16	1.73 ± 0.04	2.57 ± 0.06	1.79	2.25E-06
PARP8	1.72 ± 0.04	3.22 ± 0.07	2.82	2.86E-17
PARP9	2.21 ± 0.04	5.14 ± 0.1	7.64	1.28E-61
PDCD1	-4.48 ± 0.09	-1.99 ± 0.13	5.63	5.16E-45
PDCD1LG2	-3.57 ± 0.06	1.47 ± 0.13	32.86	8.00E-178
PIK3AP1	-0.8 ± 0.05	2.56 ± 0.11	10.21	7.66E-80
PIK3CB	3.54 ± 0.04	3.49 ± 0.07	0.96	7.57E-01
PIK3CG	-3.92 ± 0.06	-0.02 ± 0.12	14.91	2.63E-107
PLA2G12A	3.14 ± 0.03	4.42 ± 0.06	2.42	5.35E-13
PLA2G4A	-0.29 ± 0.04	2.76 ± 0.1	8.29	1.63E-66
PLA2G5	2.25 ± 0.04	4.37 ± 0.21	4.33	7.45E-33
PLA2R1	-0.16 ± 0.06	-0.29 ± 0.15	0.91	4.64E-01
PLB1	0.69 ± 0.04	2.28 ± 0.1	3.01	2.71E-19
PLBD1	-0.6 ± 0.04	2.45 ± 0.12	8.30	1.30E-66
PLCG2	0.85 ± 0.04	2.05 ± 0.09	2.29	1.67E-11
PLD1	1.16 ± 0.05	1.33 ± 0.1	1.13	3.27E-01
PLD4	1.68 ± 0.05	4.03 ± 0.13	5.09	4.38E-40
PPP2R1B	2.32 ± 0.04	3 ± 0.06	1.60	1.37E-04
PRKCD	2.54 ± 0.04	3.17 ± 0.09	1.55	3.51E-04
PSMA1	5.66 ± 0.04	6.97 ± 0.05	2.48	1.45E-13
PSMA4	5.6 ± 0.04	7.19 ± 0.05	3.00	3.33E-19
PSMC1	5.59 ± 0.04	6.8 ± 0.05	2.31	9.20E-12
PSMC2	4.5 ± 0.04	6.58 ± 0.05	4.22	9.98E-32
PSMD5	2.59 ± 0.04	3.75 ± 0.07	2.24	5.35E-11

PSME2	5.5 ± 0.04	7.23 ± 0.07	3.32	1.55E-22
PSTPIP1	1.21 ± 0.04	1.96 ± 0.11	1.69	2.03E-05
PTAFR	-0.86 ± 0.05	2.66 ± 0.11	11.44	1.18E-87
PTCD3	4.36 ± 0.04	5.04 ± 0.06	1.61	1.13E-04
PTPN2	3.37 ± 0.04	4.17 ± 0.06	1.75	5.43E-06
PTPN22	-2.34 ± 0.04	-0.61 ± 0.1	3.31	1.76E-22
PTPN6	2.01 ± 0.04	4.47 ± 0.08	5.49	8.67E-44
PTPRC	0.44 ± 0.05	2.91 ± 0.12	5.56	2.39E-44
RAC2	0.51 ± 0.05	3.65 ± 0.1	8.79	3.84E-70
RHOA	6.82 ± 0.04	8.78 ± 0.05	3.88	2.18E-28
RHOH	-2.83 ± 0.07	1.02 ± 0.11	14.39	1.49E-104
RIPK3	-0.63 ± 0.04	0.98 ± 0.09	3.07	6.81E-20
RNASEL	0.49 ± 0.03	1.87 ± 0.07	2.61	5.10E-15
RNF135	1.57 ± 0.03	3.72 ± 0.08	4.44	6.45E-34
RNLS	1.01 ± 0.03	1.24 ± 0.1	1.17	2.06E-01
RPS6KA1	2.63 ± 0.05	4.15 ± 0.07	2.87	9.79E-18
RPS6KA3	2.27 ± 0.04	3.31 ± 0.07	2.05	4.69E-09
RSAD2	0.74 ± 0.05	2.78 ± 0.16	4.11	1.03E-30
SAMHD1	2.9 ± 0.04	4.88 ± 0.07	3.94	5.73E-29
SDC2	4.21 ± 0.04	5.59 ± 0.11	2.61	6.10E-15
SEC22B	3.08 ± 0.03	4.18 ± 0.04	2.15	4.93E-10
SEC23IP	1.83 ± 0.04	3.11 ± 0.07	2.42	6.57E-13
SELL	0.23 ± 0.04	2.12 ± 0.15	3.71	1.27E-26
SERPINA1	1.57 ± 0.05	5.51 ± 0.14	15.44	4.78E-110
SERPINB2	-5.88 ± 0.09	-5.12 ± 0.34	1.69	1.85E-05
SHC1	3.81 ± 0.04	5.46 ± 0.09	3.13	1.42E-20
SIGLEC11	-3.09 ± 0.06	-0.59 ± 0.12	5.63	5.51E-45
SIGLEC12	-7.01 ± 0.09	-3.68 ± 0.26	10.00	1.86E-78
SLAMF7	-4.38 ± 0.07	-0.92 ± 0.15	11.00	6.60E-85
SLC4A7	0.71 ± 0.04	2.46 ± 0.08	3.36	5.13E-23
SNAP23	3.56 ± 0.03	4.82 ± 0.05	2.40	1.06E-12
SP100	2.49 ± 0.04	4.8 ± 0.1	4.95	8.50E-39
STAT1	3.63 ± 0.04	5.92 ± 0.09	4.86	5.37E-38
STAT2	5.27 ± 0.05	5.62 ± 0.09	1.27	4.92E-02
STAT4	1.1 ± 0.06	-1.05 ± 0.12	0.23	6.47E-34
STAT5A	1.64 ± 0.04	3.23 ± 0.08	3.01	2.70E-19
STOM	5.36 ± 0.04	6.07 ± 0.1	1.63	6.30E-05
SULT1A1	4.5 ± 0.04	3.42 ± 0.12	0.48	1.48E-09
SULT1C2	-3.1 ± 0.07	-2.28 ± 0.22	1.77	3.55E-06
SYK	-0.66 ± 0.05	2.73 ± 0.1	10.46	1.86E-81
TANK	3.65 ± 0.03	5.25 ± 0.06	3.04	1.27E-19
TBK1	2.73 ± 0.04	3.55 ± 0.05	1.77	3.57E-06
TFG	5.03 ± 0.04	6.42 ± 0.05	2.62	4.18E-15
TICAM2	-0.12 ± 0.03	1.47 ± 0.14	2.99	4.10E-19
TLR1	-0.99 ± 0.05	2.62 ± 0.1	12.26	1.29E-92
TLR10	-1.74 ± 0.05	0.16 ± 0.14	3.71	1.16E-26
TLR2	0.13 ± 0.05	2.81 ± 0.11	6.40	1.30E-51
TLR3	-1.77 ± 0.04	1.65 ± 0.11	10.72	3.56E-83
TLR5	-0.95 ± 0.04	0.94 ± 0.11	3.70	1.74E-26
TLR6	-2.44 ± 0.05	-0.16 ± 0.11	4.85	6.88E-38
TLR7	-2.48 ± 0.06	1.46 ± 0.12	15.30	2.52E-109
TLR8	-4.75 ± 0.07	-0.41 ± 0.15	20.18	3.44E-132
TMED7-TICAM2	-4.97 ± 0.11	-0.52 ± 0.18	21.88	2.76E-139
TNF	-4.89 ± 0.09	-0.42 ± 0.16	22.13	2.51E-140
TNFAIP3	0.21 ± 0.04	2.45 ± 0.09	4.73	1.10E-36
TNFRSF11B	-0.6 ± 0.06	2.47 ± 0.14	8.44	1.39E-67
TNFRSF14	3.66 ± 0.04	4.49 ± 0.08	1.77	2.97E-06
TNFRSF8	-3.76 ± 0.1	-1.77 ± 0.15	3.97	2.59E-29
TNFSF13	3.84 ± 0.04	4.63 ± 0.1	1.73	7.81E-06
TNFSF13B	0.12 ± 0.04	3.2 ± 0.11	8.49	5.62E-68
TNFSF4	-0.61 ± 0.04	0.22 ± 0.11	1.78	2.94E-06
TNFSF8	-4.02 ± 0.09	0.96 ± 0.13	31.50	1.28E-173
TREM2	1.79 ± 0.06	6.71 ± 0.1	30.30	8.83E-170
TREML1	-1.25 ± 0.06	1.1 ± 0.12	5.10	3.15E-40
TREML2	-6.93 ± 0.07	-4.24 ± 0.22	6.48	2.66E-52
TRIM14	1.28 ± 0.04	3.68 ± 0.09	5.28	8.27E-42
TRIM21	1.15 ± 0.03	3.87 ± 0.08	6.56	5.47E-53

TRIM22	2.64 ± 0.04	5.17 ± 0.12	5.77	3.33E-46
TRIM25	2.37 ± 0.04	3.66 ± 0.08	2.45	2.58E-13
TRIM34	-0.93 ± 0.04	2.25 ± 0.11	9.04	6.68E-72
TRIM38	-1.25 ± 0.03	0.92 ± 0.1	4.50	1.75E-34
TRIM5	0.26 ± 0.04	3.37 ± 0.09	8.62	6.18E-69
TRIM6	-2.32 ± 0.04	0.72 ± 0.12	8.19	9.98E-66
UBA3	4.3 ± 0.04	5.54 ± 0.05	2.36	2.36E-12
UGP2	5.96 ± 0.04	6.09 ± 0.06	1.10	4.57E-01
VAMP3	5.11 ± 0.04	6.4 ± 0.06	2.44	3.33E-13
VAMP8	2.43 ± 0.04	5.98 ± 0.1	11.69	3.38E-89
WDR11	3.78 ± 0.04	3.7 ± 0.07	0.95	6.46E-01
XAF1	3.57 ± 0.04	3.9 ± 0.17	1.25	6.77E-02
YWHAZ	7.36 ± 0.04	8.1 ± 0.05	1.67	2.91E-05

Table S3. Using the multivariate Cox proportional hazard model, TGFB2 methylation correlated genes with a significant prognostic impact on OS in GBM patients.

	Gene 2 mRNA		TGFB2 methylation		TGFB1 methylation		TGFB3 methylation		MGMT methylation	
Gene 2	HR(95% CI)	P-val	HR(95% CI)	P-val	HR(95% CI)	P-val	HR(95% CI)	P-val	HR(95% CI)	P-val
NUDT12	0.543 (0.364-0.811)	0.003	0.084 (0.003-2.769)	0.165	0.448 (0.209-0.958)	0.038	1.046 (0.545-2.004)	0.893	0.402 (0.214-0.756)	0.005
SNAP23	0.411 (0.218-0.774)	0.006	0.072 (0.002-3.314)	0.178	0.543 (0.261-1.131)	0.103	0.82 (0.435-1.545)	0.538	0.351 (0.186-0.661)	0.001
HIF1A	0.591 (0.4-0.873)	0.008	0.092 (0.002-5.03)	0.242	0.528 (0.247-1.129)	0.099	0.719 (0.37-1.398)	0.331	0.353 (0.185-0.674)	0.002
UBA3	0.431 (0.227-0.82)	0.01	0.117 (0.003-4.709)	0.255	0.486 (0.227-1.04)	0.063	0.931 (0.49-1.768)	0.827	0.362 (0.193-0.68)	0.002
BCL10	0.353 (0.16-0.776)	0.01	0.069 (0.002-2.881)	0.16	0.532 (0.247-1.144)	0.106	0.697 (0.353-1.374)	0.297	0.279 (0.139-0.562)	0
TRIM5	0.607 (0.413-0.892)	0.011	0.016 (0-0.919)	0.045	0.731 (0.333-1.605)	0.435	0.629 (0.309-1.279)	0.2	0.403 (0.217-0.749)	0.004
TRIM21	0.571 (0.368-0.886)	0.012	0.027 (0.001-1.316)	0.069	0.592 (0.286-1.225)	0.158	0.666 (0.34-1.303)	0.235	0.328 (0.171-0.63)	0.001
IFIT3	0.742 (0.587-0.939)	0.013	0.044 (0.001-2.884)	0.143	0.541 (0.259-1.13)	0.102	0.779 (0.414-1.465)	0.438	0.398 (0.215-0.738)	0.003
CAPZA1	0.471 (0.259-0.858)	0.014	0.072 (0.001-3.601)	0.187	0.546 (0.258-1.156)	0.114	0.81 (0.426-1.539)	0.52	0.351 (0.185-0.669)	0.001
PARP9	0.646 (0.452-0.925)	0.017	0.032 (0-2.073)	0.106	0.598 (0.285-1.255)	0.174	0.676 (0.343-1.331)	0.257	0.368 (0.196-0.692)	0.002
TRIM22	0.75 (0.591-0.951)	0.018	0.035 (0.001-2.259)	0.115	0.579 (0.275-1.217)	0.149	0.691 (0.352-1.357)	0.283	0.395 (0.213-0.734)	0.003
ITCH	0.385 (0.172-0.866)	0.021	0.129 (0.003-5.671)	0.289	0.44 (0.199-0.971)	0.042	0.782 (0.402-1.522)	0.469	0.43 (0.232-0.794)	0.007
STAT1	0.656 (0.457-0.94)	0.022	0.038 (0.001-2.246)	0.116	0.507 (0.24-1.073)	0.076	0.709 (0.367-1.371)	0.307	0.417 (0.225-0.771)	0.005
MALT1	1.997 (1.1-3.625)	0.023	0.067 (0.002-2.658)	0.15	0.623 (0.284-1.368)	0.239	1.041 (0.54-2.008)	0.904	0.452 (0.243-0.841)	0.012
RSAD2	0.835 (0.714-0.976)	0.024	0.04 (0.001-2.51)	0.127	0.537 (0.255-1.129)	0.101	0.807 (0.427-1.526)	0.51	0.42 (0.227-0.778)	0.006
PSMA4	0.54 (0.307-0.948)	0.032	0.136 (0.003-5.819)	0.298	0.494 (0.234-1.043)	0.064	0.937 (0.497-1.768)	0.842	0.349 (0.183-0.667)	0.001
IFIH1	0.754 (0.58-0.981)	0.035	0.036 (0.001-2.097)	0.109	0.568 (0.266-1.209)	0.142	0.709 (0.355-1.416)	0.329	0.379 (0.203-0.711)	0.002
IRAK4	0.586 (0.354-0.97)	0.038	0.066 (0.002-2.823)	0.156	0.499 (0.236-1.054)	0.068	0.811 (0.419-1.571)	0.535	0.39 (0.209-0.729)	0.003
HLA-DRB1	0.818 (0.67-0.997)	0.047	0.032 (0.001-1.915)	0.099	0.503 (0.234-1.08)	0.078	0.96 (0.507-1.816)	0.9	0.349 (0.184-0.665)	0.001
IFI35	0.772 (0.597-0.999)	0.049	0.035 (0.001-1.747)	0.093	0.568 (0.27-1.193)	0.135	0.774 (0.402-1.493)	0.445	0.355 (0.186-0.676)	0.002

	Age at Diagnosis		Male relative to Female		TGFB2 methylation x Age interaction		Male x Age interaction		Univariate Gene 2 mRNA	
Gene 2	HR(95% CI)	P-val	HR(95% CI)	P-val	HR(95% CI)	P-val	HR(95% CI)	P-val	HR(95% CI)	P-val
NUDT12	1.06 (1.025-1.097)	0.001	16.172 (0.689-379.39)	0.084	1.034 (0.979-1.093)	0.234	0.956 (0.91-1.005)	0.075	0.616 (0.424-0.896)	0.011
SNAP23	1.058 (1.022-1.094)	0.001	8.571 (0.405-181.501)	0.168	1.034 (0.973-1.099)	0.282	0.965 (0.92-1.012)	0.147	0.629 (0.357-1.108)	0.108
HIF1A	1.052 (1.017-1.088)	0.003	13.064 (0.527-324.061)	0.117	1.034 (0.97-1.102)	0.303	0.958 (0.911-1.008)	0.098	0.65 (0.46-0.917)	0.014
UBA3	1.052 (1.018-1.087)	0.002	5.851 (0.25-137.162)	0.272	1.028 (0.969-1.09)	0.364	0.973 (0.926-1.022)	0.27	0.555 (0.305-1.011)	0.054
BCL10	1.051 (1.016-1.086)	0.004	6.909 (0.27-176.832)	0.243	1.032 (0.973-1.095)	0.289	0.969 (0.921-1.019)	0.224	0.791 (0.427-1.466)	0.457
TRIM5	1.059 (1.023-1.096)	0.001	27.591 (1.049-725.755)	0.047	1.057 (0.992-1.127)	0.085	0.95 (0.903-0.999)	0.046	0.901 (0.673-1.207)	0.486
TRIM21	1.061 (1.024-1.1)	0.001	32.107 (1.134-908.805)	0.042	1.052 (0.989-1.119)	0.111	0.948 (0.9-0.998)	0.042	0.898 (0.632-1.277)	0.55
IFIT3	1.06 (1.024-1.099)	0.001	31.473 (1.155-857.778)	0.041	1.044 (0.977-1.117)	0.202	0.948 (0.9-0.997)	0.04	0.817 (0.663-1.006)	0.058
CAPZA1	1.055 (1.02-1.091)	0.002	8.095 (0.331-198.015)	0.2	1.033 (0.971-1.1)	0.303	0.966 (0.919-1.016)	0.177	0.684 (0.393-1.191)	0.18
PARP9	1.057 (1.02-1.094)	0.002	28.789 (1.051-788.577)	0.047	1.047 (0.98-1.119)	0.175	0.949 (0.902-0.999)	0.046	0.814 (0.612-1.082)	0.156
TRIM22	1.061 (1.024-1.098)	0.001	21.512 (0.85-544.293)	0.063	1.048 (0.981-1.119)	0.168	0.952 (0.906-1.001)	0.057	0.89 (0.722-1.096)	0.272
ITCH	1.05 (1.016-1.084)	0.003	5.966 (0.26-136.987)	0.264	1.029 (0.969-1.092)	0.352	0.972 (0.925-1.021)	0.25	0.448 (0.213-0.942)	0.034

STAT1	1.058 (1.022-1.096)	0.002	29.517 (1.059-822.538)	0.046	1.048 (0.982-1.118)	0.159	0.949 (0.901-0.999)	0.046	0.763 (0.56-1.041)	0.088
MALT1	1.068 (1.03-1.107)	0	26.051 (0.928-731.063)	0.055	1.041 (0.982-1.103)	0.18	0.949 (0.9-1)	0.048	1.461 (0.89-2.396)	0.134
RSAD2	1.059 (1.023-1.097)	0.001	27.189 (1.02-725.097)	0.049	1.046 (0.979-1.117)	0.185	0.95 (0.903-1)	0.049	0.89 (0.773-1.025)	0.107
PSMA4	1.057 (1.022-1.093)	0.001	5.998 (0.246-146.137)	0.271	1.024 (0.964-1.088)	0.443	0.972 (0.925-1.022)	0.268	0.826 (0.511-1.334)	0.434
IFIH1	1.058 (1.022-1.097)	0.002	29.745 (1.065-830.476)	0.046	1.045 (0.98-1.115)	0.177	0.949 (0.901-0.999)	0.044	0.883 (0.717-1.088)	0.244
IRAK4	1.053 (1.018-1.088)	0.002	7.846 (0.342-180.163)	0.198	1.036 (0.976-1.1)	0.249	0.969 (0.922-1.017)	0.204	0.784 (0.514-1.195)	0.258
HLA-DRB1	1.052 (1.017-1.088)	0.003	10.79 (0.442-263.405)	0.145	1.048 (0.983-1.119)	0.152	0.963 (0.916-1.012)	0.136	0.934 (0.775-1.127)	0.478
IFI35	1.056 (1.02-1.094)	0.002	20.898 (0.798-547.52)	0.068	1.047 (0.984-1.115)	0.143	0.953 (0.906-1.003)	0.065	0.923 (0.735-1.159)	0.49

Table S4. Reactome Pathways negatively correlated with *MGMT* Methylation.

Cluster Index	Reactome Pathway	Normalized Enrichment Score					
		***MGMT* methylation**			***TGFB2* methylation**		
		All	Female	Male	Female	All	Male
92	Degradation of DVL	-2.22	-2.03	-1.70	0.98	0.76	-0.72
93	SCF-beta-TrCP mediated degradation of Emi1	-2.22	-2.01	-1.63	0.92	0.74	-0.71
94	ROS sensing by NFE2L2	-2.13	-2.04	-1.60	0.91	0.80	-0.77
95	SCF(Skp2)-mediated degradation of p27/p21	-2.27	-1.91	-1.64	1.13	0.63	-0.82
96	Autodegradation of Cdh1 by Cdh1:APC/C	-2.06	-1.95	-1.57	1.02	0.91	-0.82
97	APC/C:Cdc20 mediated degradation of Securin	-2.05	-1.99	-1.50	1.07	0.89	-0.85
98	CDK-mediated phosphorylation and removal of Cdc6	-2.01	-1.80	-1.52	1.18	0.92	-0.77
99	Cellular response to hypoxia	-1.99	-1.68	-1.82	0.90	0.75	-0.67
100	G1/S DNA Damage Checkpoints	-2.00	-1.72	-1.57	1.02	0.76	-0.75
101	Regulation of RAS by GAPs	-1.94	-1.63	-1.60	0.69	0.70	-0.73
102	Cdc20:Phospho-APC/C mediated degradation of Cyclin A	-2.04	-1.96	-1.42	1.34	0.94	-0.85
103	APC:Cdc20 mediated degradation of cell cycle proteins prior to satisfation of the cell cycle checkpoint	-2.03	-1.96	-1.43	1.42	0.93	-0.84
104	APC/C:Cdc20 mediated degradation of mitotic proteins	-2.02	-1.96	-1.42	1.58	1.03	-0.83
105	Activation of APC/C and APC/C:Cdc20 mediated degradation of mitotic proteins	-2.02	-1.96	-1.43	1.66	1.11	-0.79
106	SLBP Dependent Processing of Replication-Dependent Histone Pre-mRNAs	-2.16	-2.07	-1.38	0.77	0.71	0.93
107	SLBP independent Processing of Histone Pre-mRNAs	-2.16	-2.02	-1.28	0.75	0.76	0.82
108	PCP/CE pathway	-1.93	-1.66	-1.77	0.81	0.84	0.86
109	RUNX1 regulates transcription of genes involved in differentiation of HSCs	-1.90	-1.66	-1.57	0.95	0.88	0.81
110	Degradation of beta-catenin by the destruction complex	-1.92	-1.86	-1.30	0.94	1.04	0.92
111	Regulation of PTEN stability and activity	-1.81	-1.64	-1.35	1.10	1.08	0.76
112	Cyclin E associated events during G1/S transition	-2.09	-1.69	-1.36	1.25	0.92	0.80
113	Cyclin A:Cdk2-associated events at S phase entry	-2.06	-1.64	-1.36	1.36	1.03	0.88
114	Orc1 removal from chromatin	-2.26	-1.94	-1.67	1.28	1.10	0.89
115	The role of GTSE1 in G2/M progression after G2 checkpoint	-2.18	-1.79	-1.79	1.50	1.21	0.81
116	Assembly of the pre-replicative complex	-2.14	-1.82	-1.67	1.33	1.31	1.16

117	DNA Replication Pre-Initiation	-2.15	-1.84	-1.71	1.67	1.46	1.17
118	APC/C:Cdh1 mediated degradation of Cdc20 and other APC/C:Cdh1 targeted proteins in late mitosis/early G1	-1.96	-1.87	-1.44	1.49	1.16	0.87
119	Regulation of APC/C activators between G1/S and early anaphase	-1.96	-1.85	-1.38	1.57	1.14	0.76
120	Switching of origins to a post-replicative state	-1.98	-1.72	-1.49	1.53	1.30	0.99
121	APC/C-mediated degradation of cell cycle proteins	-1.88	-1.84	-1.32	1.81	1.19	0.76
122	Regulation of mitotic cell cycle	-1.88	-1.84	-1.32	1.81	1.19	0.76

Table S5. Upregulation of genes in GBM tumors correlated with *MGMT* methylation.

Gene	Normal Expression (Mean Log2 TPM ± SEM)	Tumor Expression (Mean Log2 TPM ± SEM)	Fold change (Tumor/Normal)	P-value
ANAPC11	6.39 ± 0.04	8.14 ± 0.05	3.35	6.41E-33
ANAPC16	5.15 ± 0.04	5.87 ± 0.05	1.66	5.83E-07
AP2S1	6.19 ± 0.04	7.48 ± 0.05	2.45	8.27E-19
CA9	-1.21 ± 0.1	3.34 ± 0.24	23.36	2.71E-211
CHEK2	0.07 ± 0.04	2.77 ± 0.07	6.47	7.72E-76
CITED2	3.29 ± 0.06	4.95 ± 0.08	3.16	5.80E-30
CLTA	6.86 ± 0.04	7.56 ± 0.05	1.63	1.36E-06
CTBP2	3.68 ± 0.04	4.32 ± 0.06	1.56	9.71E-06
LSM10	4.78 ± 0.03	6.4 ± 0.06	3.07	1.33E-28
MNAT1	3.8 ± 0.03	4.38 ± 0.06	1.50	7.02E-05
NCBP2	5 ± 0.03	6.25 ± 0.06	2.38	9.98E-18
PFN1	6.46 ± 0.04	9 ± 0.05	5.83	8.00E-68
POLE4	5.28 ± 0.04	6.41 ± 0.1	2.20	7.76E-15
PSMA2	5.13 ± 0.04	7.57 ± 0.06	5.40	2.67E-62
PSMA3	5.3 ± 0.04	6.56 ± 0.05	2.40	4.26E-18
PSMA4	5.6 ± 0.04	7.19 ± 0.05	3.00	1.67E-27
PSMA5	4.1 ± 0.04	6.13 ± 0.05	4.08	7.94E-44
PSMB10	5.58 ± 0.04	6.33 ± 0.05	1.69	2.49E-07
PSMB3	5.84 ± 0.04	7.91 ± 0.04	4.21	9.61E-46
PSMB4	7.06 ± 0.04	8.55 ± 0.04	2.81	1.74E-24
PSMB6	5.94 ± 0.04	7.43 ± 0.04	2.81	1.96E-24
PSMB7	6.34 ± 0.04	7.64 ± 0.05	2.47	4.72E-19
PSMC1	5.59 ± 0.04	6.8 ± 0.05	2.31	1.30E-16
PSMC4	4.99 ± 0.04	6.71 ± 0.05	3.29	5.07E-32
PSMD14	4.39 ± 0.04	6.08 ± 0.05	3.22	6.62E-31
PSMD4	6.74 ± 0.04	7.41 ± 0.04	1.59	4.12E-06
RBX1	5.37 ± 0.04	6.25 ± 0.06	1.85	1.36E-09
RPA1	3.94 ± 0.04	6.01 ± 0.06	4.20	1.09E-45
RPA2	4.32 ± 0.04	6.15 ± 0.06	3.57	3.48E-36
RPS27A	9.12 ± 0.04	10.18 ± 0.06	2.09	3.48E-13
SKP1	8.5 ± 0.04	9.74 ± 0.06	2.37	1.53E-17
SNRPE	5.26 ± 0.04	7.42 ± 0.09	4.47	1.78E-49
SNRPF	3.73 ± 0.03	5.44 ± 0.04	3.26	1.45E-31
SNRPG	4.99 ± 0.03	7.82 ± 0.06	7.12	1.39E-83
TUBA1B	8.98 ± 0.05	10.94 ± 0.07	3.88	7.31E-41
TUBB6	3.6 ± 0.04	5.95 ± 0.11	5.11	2.27E-58
UBB	10.07 ± 0.04	11.33 ± 0.05	2.39	6.30E-18
WNT5A	-0.4 ± 0.04	2.69 ± 0.1	8.50	5.84E-99
WTIP	1.78 ± 0.03	2.4 ± 0.11	1.54	2.11E-05

TableS6. Cox proportional hazards model for genes that exhibited significant prognostic impact of MGMT methylation and significantly upregulated in tumors.

Gene 2	Gene 2 mRNA HR(95% CI)	P-val	TGFB2 methylation HR(95% CI)	P-val	TGFB1 methylation HR(95% CI)	P-val	TGFB3 methylation HR(95% CI)	P-val	MGMT methylation HR(95% CI)	P-val
SNRPG	0.694 (0.479-1.006)	0.054	0.079 (0.002-3.108)	0.175	0.521 (0.245-1.108)	0.09	0.95 (0.5-1.804)	0.875	0.339 (0.175-0.657)	0.001
PSMA4	0.54 (0.307-0.948)	0.032	0.136 (0.003-5.819)	0.298	0.494 (0.234-1.043)	0.064	0.937 (0.497-1.768)	0.842	0.349 (0.183-0.667)	0.001
PSMB10	0.742 (0.501-1.099)	0.137	0.038 (0.001-1.729)	0.093	0.545 (0.258-1.152)	0.112	0.906 (0.481-1.705)	0.759	0.357 (0.185-0.69)	0.002
PSMA5	0.629 (0.359-1.102)	0.105	0.067 (0.002-2.655)	0.15	0.543 (0.253-1.165)	0.117	0.882 (0.458-1.698)	0.707	0.358 (0.186-0.691)	0.002
PSMD14	0.567 (0.308-1.041)	0.067	0.076 (0.002-2.621)	0.153	0.525 (0.246-1.121)	0.096	0.99 (0.518-1.89)	0.975	0.358 (0.187-0.687)	0.002
CTBP2	2.015 (0.952-4.262)	0.067	0.041 (0.001-2.04)	0.109	0.544 (0.255-1.163)	0.117	0.809 (0.418-1.566)	0.53	0.373 (0.196-0.708)	0.003
RBX1	0.796 (0.575-1.102)	0.17	0.073 (0.002-2.963)	0.166	0.537 (0.251-1.147)	0.108	0.914 (0.48-1.742)	0.785	0.362 (0.186-0.703)	0.003
UBB	0.671 (0.38-1.185)	0.169	0.055 (0.001-2.407)	0.133	0.485 (0.225-1.046)	0.065	1.04 (0.539-2.008)	0.907	0.382 (0.202-0.725)	0.003
SKP1	0.723 (0.45-1.161)	0.18	0.067 (0.002-2.713)	0.152	0.483 (0.224-1.042)	0.064	0.91 (0.477-1.738)	0.775	0.372 (0.193-0.715)	0.003
RPS27A	0.73 (0.464-1.151)	0.175	0.058 (0.001-2.341)	0.131	0.503 (0.233-1.085)	0.08	0.922 (0.482-1.764)	0.806	0.349 (0.176-0.692)	0.003
CITED2	0.742 (0.498-1.105)	0.142	0.045 (0.001-2.18)	0.117	0.534 (0.25-1.142)	0.106	0.774 (0.386-1.555)	0.472	0.389 (0.207-0.731)	0.003
PSMB3	0.705 (0.435-1.143)	0.157	0.057 (0.001-2.212)	0.125	0.521 (0.243-1.116)	0.093	0.98 (0.514-1.867)	0.95	0.358 (0.184-0.698)	0.003
AP2S1	0.787 (0.495-1.252)	0.312	0.057 (0.001-2.408)	0.133	0.501 (0.233-1.078)	0.077	0.91 (0.476-1.738)	0.774	0.377 (0.193-0.735)	0.004
PSMA3	0.76 (0.471-1.227)	0.262	0.056 (0.001-2.599)	0.141	0.554 (0.259-1.182)	0.127	0.861 (0.448-1.657)	0.655	0.37 (0.189-0.726)	0.004
PSMA2	0.772 (0.485-1.23)	0.277	0.051 (0.001-2.16)	0.119	0.508 (0.237-1.093)	0.083	0.943 (0.495-1.798)	0.86	0.377 (0.195-0.732)	0.004
PFN1	0.682 (0.366-1.268)	0.226	0.052 (0.001-2.444)	0.132	0.548 (0.257-1.169)	0.12	0.903 (0.475-1.716)	0.755	0.36 (0.181-0.715)	0.004
RPA1	0.595 (0.273-1.299)	0.193	0.049 (0.001-2.529)	0.134	0.535 (0.248-1.151)	0.109	0.913 (0.476-1.748)	0.783	0.398 (0.212-0.746)	0.004
PSMC4	0.63 (0.322-1.231)	0.176	0.052 (0.001-2.049)	0.115	0.524 (0.244-1.124)	0.097	0.83 (0.423-1.63)	0.589	0.403 (0.216-0.752)	0.004
LSM10	0.824 (0.555-1.224)	0.338	0.057 (0.001-2.407)	0.133	0.549 (0.253-1.192)	0.129	0.89 (0.459-1.726)	0.73	0.385 (0.199-0.742)	0.004
PSMB6	0.705 (0.368-1.349)	0.291	0.055 (0.001-2.222)	0.124	0.521 (0.242-1.12)	0.095	0.951 (0.499-1.814)	0.879	0.377 (0.194-0.734)	0.004
ANAPC16	0.853 (0.56-1.301)	0.461	0.058 (0.001-2.585)	0.141	0.516 (0.241-1.106)	0.089	0.961 (0.505-1.826)	0.902	0.398 (0.208-0.763)	0.005
SNRPE	0.899 (0.676-1.197)	0.467	0.047 (0.001-2.592)	0.135	0.518 (0.241-1.114)	0.092	0.959 (0.506-1.82)	0.899	0.409 (0.218-0.769)	0.005
PSMB7	0.805 (0.399-1.625)	0.545	0.051 (0.001-2.247)	0.123	0.535 (0.248-1.158)	0.113	1.006 (0.511-1.982)	0.986	0.417 (0.223-0.78)	0.006
RPA2	0.863 (0.527-1.414)	0.559	0.05 (0.001-2.481)	0.133	0.527 (0.246-1.13)	0.1	0.969 (0.507-1.851)	0.925	0.41 (0.217-0.776)	0.006
ANAPC11	0.887 (0.596-1.319)	0.553	0.059 (0.001-2.574)	0.142	0.52 (0.241-1.119)	0.094	0.941 (0.493-1.796)	0.855	0.404 (0.211-0.773)	0.006
WTIP	1.007 (0.753-1.347)	0.96	0.058 (0.001-2.9)	0.153	0.526 (0.245-1.133)	0.101	0.951 (0.474-1.909)	0.888	0.429 (0.231-0.797)	0.007
MNAT1	0.862 (0.446-1.664)	0.658	0.07 (0.001-3.692)	0.188	0.546 (0.25-1.192)	0.129	0.877 (0.426-1.806)	0.721	0.416 (0.22-0.786)	0.007
CHEK2	0.932 (0.594-1.463)	0.76	0.062 (0.001-2.898)	0.156	0.525 (0.244-1.129)	0.099	0.961 (0.5-1.85)	0.906	0.421 (0.225-0.791)	0.007
PSMB4	0.87 (0.497-1.522)	0.625	0.058 (0.001-2.554)	0.14	0.529 (0.246-1.137)	0.103	0.937 (0.491-1.786)	0.843	0.406 (0.211-0.783)	0.007
CLTA	0.976 (0.604-1.579)	0.923	0.056 (0.001-2.644)	0.143	0.527 (0.245-1.136)	0.102	0.949 (0.495-1.818)	0.874	0.427 (0.229-0.798)	0.008
NCBP2	1.108 (0.545-2.252)	0.776	0.059 (0.001-2.819)	0.152	0.523 (0.244-1.123)	0.097	0.933 (0.488-1.784)	0.834	0.431 (0.232-0.8)	0.008
WNT5A	0.821 (0.629-1.073)	0.149	0.051 (0.001-3.022)	0.153	0.504 (0.232-1.095)	0.083	1.066 (0.549-2.071)	0.85	0.441 (0.239-0.814)	0.009
SNRPF	0.995 (0.549-1.8)	0.986	0.057 (0.001-2.688)	0.145	0.526 (0.244-1.134)	0.101	0.945 (0.493-1.812)	0.866	0.429 (0.227-0.81)	0.009

Gene 2	Age at Diagnosis HR(95% CI)	P-val	Male relative to Female HR(95% CI)	P-val	TGFB2 methylation x Age interaction HR(95% CI)	P-val	Male x Age interaction HR(95% CI)	P-val	Univariate Gene 2 mRNA HR(95% CI)	P-val
SNRPG	1.053 (1.018-1.089)	0.003	6.766 (0.284-161.068)	0.237	1.034 (0.975-1.096)	0.263	0.969 (0.922-1.019)	0.218	0.909 (0.659-1.254)	0.562
PSMA4	1.057 (1.022-1.093)	0.001	5.998 (0.246-146.137)	0.271	1.024 (0.964-1.088)	0.443	0.972 (0.925-1.022)	0.268	0.826 (0.511-1.334)	0.434
PSMB10	1.053 (1.017-1.089)	0.003	11.878 (0.483-291.939)	0.13	1.048 (0.986-1.114)	0.129	0.961 (0.914-1.01)	0.119	0.985 (0.69-1.406)	0.933
PSMA5	1.052 (1.017-1.088)	0.003	8.183 (0.354-189.234)	0.19	1.036 (0.977-1.099)	0.234	0.967 (0.921-1.016)	0.184	0.876 (0.553-1.386)	0.571
PSMD14	1.048 (1.014-1.084)	0.006	5.763 (0.238-139.425)	0.281	1.034 (0.977-1.094)	0.25	0.973 (0.925-1.023)	0.277	0.77 (0.458-1.294)	0.323
CTBP2	1.063 (1.026-1.101)	0.001	20.574 (0.704-601.123)	0.079	1.045 (0.982-1.112)	0.165	0.953 (0.904-1.004)	0.072	0.958 (0.489-1.876)	0.9

RBX1	1.051 (1.017-1.087)	0.003	7.851 (0.331-186.406)	0.202	1.038 (0.978-1.1)	0.218	0.967 (0.92-1.016)	0.178	0.918 (0.679-1.242)	0.58
UBB	1.051 (1.016-1.087)	0.004	8.293 (0.354-194.066)	0.189	1.041 (0.98-1.105)	0.191	0.967 (0.92-1.016)	0.182	0.904 (0.546-1.495)	0.694
SKP1	1.048 (1.014-1.084)	0.006	6.085 (0.253-146.165)	0.266	1.038 (0.979-1.101)	0.213	0.971 (0.924-1.021)	0.25	0.914 (0.6-1.394)	0.677
RPS27A	1.049 (1.014-1.084)	0.006	7.338 (0.317-170.055)	0.214	1.04 (0.98-1.103)	0.193	0.968 (0.922-1.017)	0.196	0.949 (0.645-1.396)	0.789
CITED2	1.051 (1.017-1.087)	0.003	10.041 (0.413-244.01)	0.156	1.047 (0.984-1.114)	0.146	0.963 (0.916-1.012)	0.138	0.919 (0.651-1.297)	0.629
PSMB3	1.052 (1.018-1.088)	0.003	8.289 (0.352-195.251)	0.19	1.04 (0.982-1.102)	0.179	0.966 (0.92-1.015)	0.174	0.998 (0.663-1.501)	0.992
AP2S1	1.05 (1.016-1.086)	0.004	7.482 (0.306-183.067)	0.217	1.042 (0.982-1.106)	0.176	0.967 (0.92-1.017)	0.195	1.066 (0.719-1.58)	0.751
PSMA3	1.055 (1.02-1.092)	0.002	12.031 (0.49-295.559)	0.128	1.04 (0.979-1.106)	0.204	0.961 (0.914-1.01)	0.116	1.063 (0.703-1.607)	0.773
PSMA2	1.05 (1.016-1.086)	0.004	8.519 (0.365-198.559)	0.182	1.042 (0.982-1.105)	0.177	0.966 (0.92-1.015)	0.171	1.013 (0.675-1.521)	0.948
PFN1	1.054 (1.019-1.091)	0.002	9.56 (0.4-228.78)	0.163	1.042 (0.98-1.108)	0.186	0.964 (0.917-1.013)	0.144	1.122 (0.667-1.889)	0.664
RPA1	1.051 (1.016-1.087)	0.004	8.81 (0.377-205.885)	0.176	1.043 (0.98-1.11)	0.189	0.965 (0.919-1.014)	0.163	0.753 (0.359-1.576)	0.451
PSMC4	1.052 (1.018-1.087)	0.003	11.629 (0.508-266.167)	0.125	1.043 (0.984-1.106)	0.157	0.962 (0.916-1.01)	0.118	0.855 (0.479-1.525)	0.596
LSM10	1.049 (1.014-1.085)	0.005	8.213 (0.351-191.982)	0.19	1.041 (0.981-1.105)	0.184	0.966 (0.92-1.015)	0.173	0.938 (0.665-1.322)	0.714
PSMB6	1.053 (1.018-1.088)	0.003	10.367 (0.444-241.839)	0.146	1.041 (0.981-1.104)	0.182	0.964 (0.918-1.012)	0.143	1.067 (0.611-1.864)	0.818
ANAPC16	1.051 (1.017-1.088)	0.004	8.769 (0.371-207.398)	0.179	1.041 (0.98-1.106)	0.195	0.965 (0.919-1.014)	0.159	0.994 (0.652-1.515)	0.978
SNRPE	1.05 (1.014-1.086)	0.006	7.623 (0.302-192.338)	0.217	1.044 (0.98-1.113)	0.183	0.967 (0.92-1.017)	0.197	0.972 (0.746-1.267)	0.835
PSMB7	1.054 (1.019-1.09)	0.002	11.132 (0.446-278.052)	0.142	1.043 (0.982-1.108)	0.166	0.962 (0.915-1.011)	0.128	1.1 (0.62-1.951)	0.744
RPA2	1.053 (1.018-1.089)	0.003	10.154 (0.428-240.93)	0.151	1.042 (0.98-1.108)	0.188	0.963 (0.916-1.012)	0.134	1.112 (0.71-1.74)	0.642
ANAPC11	1.051 (1.016-1.087)	0.004	8.593 (0.355-207.934)	0.186	1.041 (0.981-1.105)	0.187	0.965 (0.918-1.015)	0.167	1.034 (0.724-1.478)	0.852
WTIP	1.053 (1.017-1.089)	0.004	9.685 (0.401-234.05)	0.162	1.041 (0.978-1.108)	0.206	0.963 (0.917-1.013)	0.143	1.028 (0.8-1.321)	0.83
MNAT1	1.053 (1.018-1.09)	0.003	10.569 (0.431-259.236)	0.149	1.038 (0.974-1.106)	0.255	0.963 (0.916-1.012)	0.133	0.881 (0.518-1.499)	0.641
CHEK2	1.052 (1.017-1.088)	0.004	8.8 (0.345-224.691)	0.188	1.04 (0.979-1.106)	0.204	0.965 (0.917-1.015)	0.162	0.921 (0.604-1.404)	0.702
PSMB4	1.051 (1.016-1.087)	0.004	8.504 (0.343-210.653)	0.191	1.041 (0.98-1.105)	0.191	0.966 (0.918-1.016)	0.174	1.089 (0.659-1.799)	0.739
CLTA	1.052 (1.017-1.088)	0.003	9.574 (0.396-231.623)	0.165	1.041 (0.98-1.107)	0.193	0.964 (0.917-1.013)	0.146	1.083 (0.671-1.748)	0.743
NCBP2	1.053 (1.018-1.089)	0.003	9.303 (0.386-224.499)	0.17	1.041 (0.979-1.107)	0.196	0.964 (0.917-1.013)	0.144	0.963 (0.543-1.709)	0.899
WNT5A	1.055 (1.02-1.092)	0.002	11.309 (0.466-274.313)	0.136	1.04 (0.975-1.11)	0.233	0.962 (0.915-1.011)	0.128	0.865 (0.687-1.089)	0.217
SNRPF	1.052 (1.017-1.089)	0.003	9.598 (0.359-256.792)	0.177	1.041 (0.979-1.107)	0.197	0.964 (0.915-1.015)	0.161	1.169 (0.683-2.002)	0.569
